# Siglec-1-targeting single domain antibodies block or enhance pathogen binding to dendritic cells

**DOI:** 10.1016/j.jbc.2026.113542

**Published:** 2026-09-09

**Authors:** Hendrik J. Brink, Alsya J. Affandi, Shirley Man, Noah C.C.T. Dekker, Enno A. Großewichtrup, Rianne G. Bouma, Negisa Seyed Toutounchi, Veronique A.L. Konijn, Joeke G.C. Stolwijk, John L. van Hamme, Katarzyna Olesek, Dijmphna A.M. Heijnen, Alexander Fish, Angeliki Moutsiopoulou, Patrick H.N. Celie, Gillian Dekkers, Raimond Heukers, Enrico Mastrobattista, Martine J. Smit, Yvette van Kooyk, Marit J. van Gils, Astrid P. Heikema, Neeltje A. Kootstra, Teunis B.H. Geijtenbeek, Joke M.M. den Haan

**Affiliations:** 1 Department of Molecular Cell Biology and Immunology, Amsterdam UMC location Vrije Universiteit Amsterdam, Amsterdam, the Netherlands; 2 Amsterdam institute for Immunology and Infectious Diseases, Amsterdam, the Netherlands; 3 Cancer Center Amsterdam, Amsterdam, the Netherlands; 4 Department of Experimental Immunology, Amsterdam UMC, University of Amsterdam, Amsterdam, the Netherlands; 5 Division of Biochemistry, The Netherlands Cancer Institute, Oncode Institute, Amsterdam, the Netherlands; 6 QVQ Holding BV, Utrecht, the Netherlands; 7 Department of Pharmaceutics, Utrecht Institute for Pharmaceutical Sciences, Utrecht University, Utrecht, the Netherlands; 8 Amsterdam Institute for Molecular and Life Sciences (AIMMS), Department of Chemistry and Pharmaceutical Sciences, Faculty of Sciences, Vrije Universiteit, Amsterdam, the Netherlands; 9 Department of Medical Microbiology and Infection Prevention, Amsterdam University Medical Center, Amsterdam, the Netherlands; 10 Leiden Centre for Applied Bioscience, University of Leiden, Leiden, the Netherlands

**Keywords:** CD169, campylobacter jejuni, liposomes, nanobody, single-domain antibody, Siglec-1, sialoadhesin, SARS-CoV-2

## Abstract

Siglec-1 (Sialoadhesin/CD169), expressed on myeloid cells, is a receptor for sialic acids that mediates adhesion to host cells and pathogens. However, certain pathogens exploit Siglec-1 binding for further dissemination and interfering with this binding may serve as a potential therapeutic intervention. Here we identified high-affinity single domain antibodies, (also known as VHHs or Nanobodies) that allo- or orthosterically modulate ligand binding of Siglec-1. VHH clone 2C2 bound directly to the ligand binding site of Siglec-1 and effectively blocked binding of a diverse set of ligands, such as ganglioside liposomes, *Campylobacter jejuni*, and SARS-CoV-2, to monocyte-derived dendritic cells and *ex vivo* Siglec-1^+^ DCs. In contrast, VHH 1B5 and 1C1 bound outside of the ligand binding site and enhanced Siglec-1 interactions with ganglioside liposomes and *C. jejuni*. Mechanistically, VHH 1B5 and 1C1 appear to interfere with the *cis*-binding sialic acids present on the Siglec-1-expressing cell surface, thereby facilitating increased *trans*-interactions with ligands. In conclusion, we have isolated VHH that bind to different sites on Siglec-1 and thereby enhance or block ligand binding to a variety of sialylated pathogens. Moreover, unlike conventional blocking antibodies targeting specific pathogens, Siglec-1 binding VHH may serve as broad-spectrum pathogen blocking agents.

Sialic acid-binding immunoglobulin-like lectin 1 receptor (Siglec-1) also known as sialoadhesin or CD169, is expressed by macrophages and dendritic cells (DCs) (1, 2, 3, 4, 5, 6, 7, 8). Siglec-1 is a large (182 kDa) type 1 membrane receptor that structurally consists of a N-terminal V-set immunoglobulin ligand binding domain, 16 C2-set immunoglobulin domains and a transmembrane domain with a short cytoplasmic tail that lacks an immunoreceptor tyrosine-based inhibitory motif typically found in other Siglecs (9, 10). Siglec-1 binds to sialic acid N-acetylneuraminic acid with a preference for α(2, 3)-linkage to O- and N-glycans on glycoproteins and glycolipids such as gangliosides.

Siglec-1 mediates adhesion to sialic acid-expressing pathogens as well as host cells such as innate lymphocytes, neutrophils, DCs and regulatory T cells (9, 11, 12, 13). Due to its large size Siglec-1 extends far away from the cell membrane, enabling *trans-*interactions with sialic acids expressed on other cells while also capable of engaging ligands in *cis*-interactions (14, 15, 16, 17). Moreover, the extended size allows the capture of circulating pathogens and extracellular vesicles in the lymph node subcapsular sinus and splenic marginal sinus which prevents further dissemination (18, 19, 20, 21, 22).

Known pathogens captured by Siglec-1 range from enveloped viruses including HIV-1, Ebola, and SARS-CoV-2 to bacteria such as *Neisseria meningitidis* and *Campylobacter jejuni* (5, 22, 23, 24, 25, 26, 27, 28, 29). In the case of HIV-1 and Ebola, Siglec-1 binds to GM1a and GM3 gangliosides that are incorporated into the viral membrane upon budding (22, 29). HIV-1 and SARS-CoV-2 have developed strategies to exploit the capture by Siglec-1 to trans-infect neighboring CD4^+^ T-cells or ACE2^+^ cells, enabling viral dissemination in the absence of a productive infection (22, 25, 30, 31). Binding to Siglec-1 routes viruses to virus-containing-compartments that prevent degradation of virus and allow subsequent transmission to interacting cells (32, 33).

*C. jejuni* is a foodborne pathogen and one of the leading causes of bacterial gastroenteritis worldwide. A very small subset of patients develop Guillain-Barré Syndrome (GBS), due to the generation of antibodies against sialylated lipooligosaccharide (LOS) structures of *C. jejuni* that cross-react with host gangliosides such as GD1a, GM1 and GQ1b on peripheral nerves, resulting in paralysis (34, 35, 36, 37). GBS-associated *C. jejuni* strains preferentially bind to and are taken up by Siglec-1 macrophages and in a mouse model this Siglec-1-mediated uptake induces peripheral nerve neuropathy *via* the generation of ganglioside targeting auto-antibodies (38, 39, 40, 41).

Considering the crucial role of Siglec-1 in capturing pathogens, our aim was to generate variable heavy domain of heavy (VHH) chain-only antibodies also known as single-domain antibodies or Nanobodies against human Siglec-1 that can modulate Siglec-1 ligand binding. VHH are small (∼15 kDa) and bind to their targets with high affinity (42). VHH typically have long CDR3 loops that, combined with a relatively large paratope and small size, enable them to bind to epitopes inaccessible to conventional antibodies (42, 43). VHH can act as conformational stabilizers, locking a target protein into a fixed state, enabling the interrogation of structure/function relationships (44, 45).

In this study, we generated high-affinity Siglec-1 targeting VHHs and characterized three clones (1B5, 1C1 and 2C2) for their capacity to bind and modulate Siglec-1 ligand interactions. VHH clones 1B5 and 1C1 bound Siglec-1 outside of the ligand binding site and have a predicted binding site on the interface between domain 1 and 2, whereas 2C2 bound directly to the ligand binding site of Siglec-1. We reveal that VHH 1B5 and 1C1 could positively modulate Siglec-1’s adhesive function, as observed by the enhancement of sialylated pathogen binding of *C. jejuni* by cell lines and primary antigen presenting cells (APCs). In contrast, 2C2 blocked Siglec-1-dependent interactions with *C. jejuni* and SARS-CoV-2. We have, therefore, generated new tools that positively or negatively regulate Siglec-1 binding to various sialylated pathogens, which will help to further decipher Siglec-1’s function and could be explored as potential therapeutics.

## Results

### Immunization strategy and selection of Siglec-1 binding VHH

To generate human Siglec-1-specific VHH two llamas were immunized with human Siglec-1 expressing cells and recombinant Siglec-1 protein (Figs. 1*A* and S1*A*). Phage-display libraries were generated and screened for binding by ELISA. Next, clones were selected for VHH production and screened as periplasmic extracts for binding to cellular and recombinant Siglec-1 by flow cytometry and ELISA, respectively (Fig. S1*B*). VHH clones were subdivided into 86 VHH families based on their CDR3 regions (similarity ≥ 80%). A selection of 22 clones were produced as recombinant protein of which 18 bound to Siglec-1 expressing BW517 cells (BWSn) by flow cytometry (Fig. S1, *C* and *D*). Next, VHH clones 1B5, 1C1 and 2C2 were selected for further characterization. Half-maximal binding concentrations were determined for binding to BWSn cells. 1B5 and 2C2 displayed half-maximal binding concentrations in the low nanomolar range of 10 ± 3 nM and 15 ± 9 nM, similar to the Siglec-1-specific monoclonal antibody (mAb) 7-239 3 ± 1 nM, while that of 1C1 was in the higher nanomolar range 170 ± 95 nM (Fig. 1*B*). We next determined the affinities of our VHH using recombinant Siglec-1 in surface plasmon resonance measurements (Fig. S1, *E* and *F*). VHH clones 1B5 and 2C2 bound with a high affinity (0.6 and 7.4 nM), while 1C1 displayed a lower affinity (21 nM). In conclusion, 18 VHH clones were identified that bind to human Siglec-1 and three clones 1B5, 1C1 and 2C2 were selected for further characterization.

**Figure 1:**
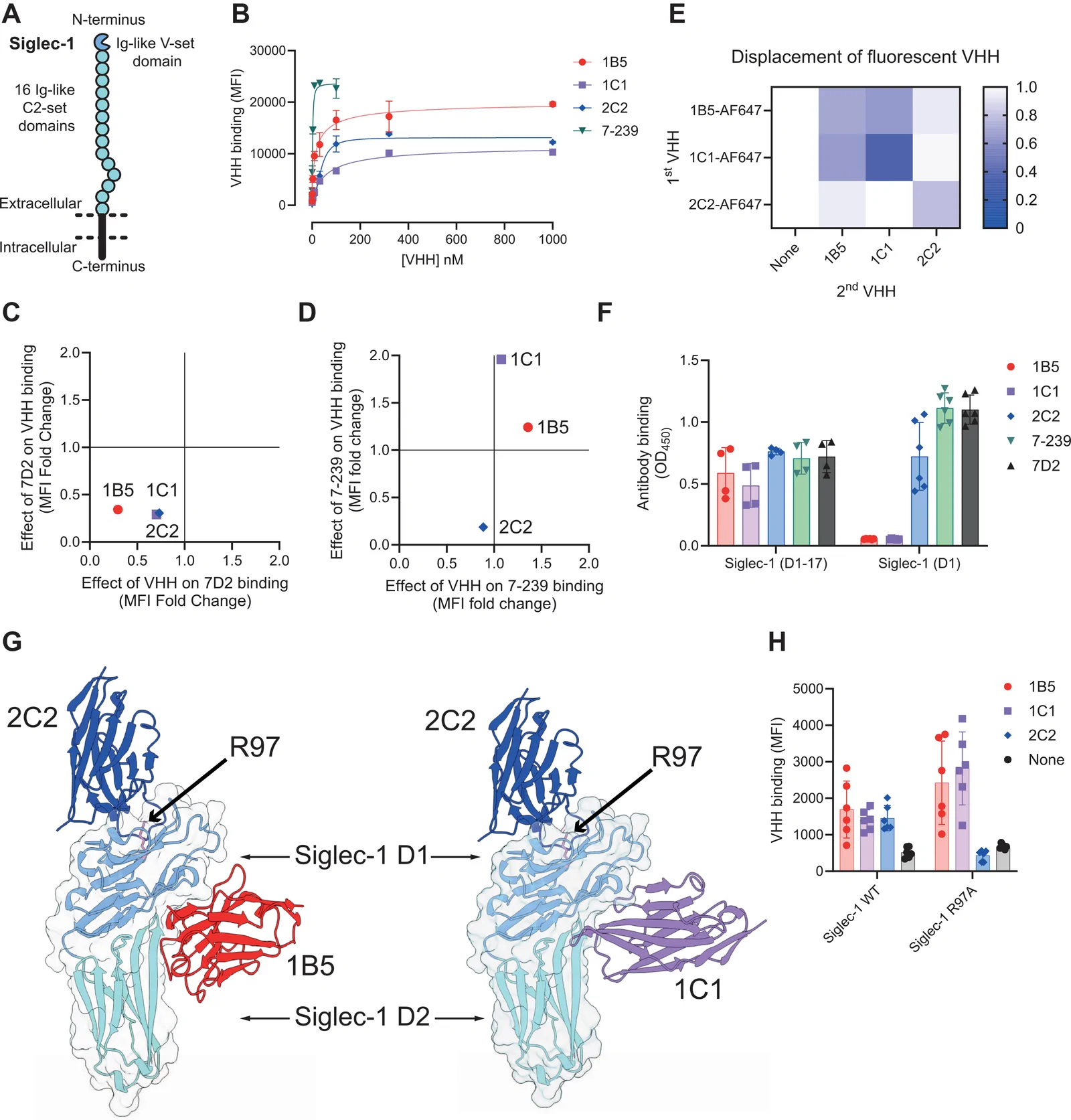
**Selection and epitope mapping of Siglec-1-binding VHH.***A*, schematic overview of Siglec-1. *B*, binding curves of selected VHH 1B5, 1C1, and 2C2 and conventional antibody 7-239 binding to BWSn cells. Geometric mean fluorescent intensity is shown. *C* and *D*, effect of preincubation with conventional antibody 7D2 or 7-239 on VHH binding to BWSn (y-axis) or the reverse, in which cells were preincubated with VHH followed by 7D2 or 7-239 binding (x-axis). Data is normalized to a no antibody/VHH control. *E*, AF647 labelled VHH binding to BWSn cells after displacement with nonlabelled VHH. Data is normalized to the no competing VHH condition. *F*, ELISA binding data of VHH and antibodies to recombinant Siglec-1-D1-17 and Siglec-1-D1. *G*, AF3 model of VHH clone 2C2 (*blue*) binding to Siglec-1 D1 superimposed with either 1B5 (*red*) or 1C1 (*purple*) binding to Siglec-1 D1-2. Siglec-1 R97 is indicated by an *arrow*. *H*, VHH binding to HEK293T cells expressing WT or R97A mutant Siglec-1. Data is shown as a representative figure of *n* = 3-4 experiments as mean ± *SD*. Siglec-1, Sialic acid-binding immunoglobulin-like lectin 1.

### Epitope mapping of Siglec-1 binding VHH

Siglec-1 is a large type 1 membrane receptor with 17 extracellular domains of which the terminal V-set domain 1 (Siglec-1-D1) is responsible for binding to sialic acids (Fig. 1*A*). Ligand binding can be blocked by mAb 7D2 and 7-239 that are known to recognize different epitopes (26, 46, 47). First, we performed blocking assays with VHH and mAb 7D2/7-239. Pre-incubation of cells with 7D2 blocked binding of all three VHH and in the reverse set up all three VHH blocked 7D2, which suggests that all three VHH bind to or near the terminal domain (Figs. 1*C* and S2*E*). In contrast, preincubating cells with 7-239 significantly increased the binding of 1B5 and 1C1, whereas 2C2 binding was efficiently blocked (Fig. 1*D*). In the reverse set up, 7-239 binding was significantly increased by preincubation with 1B5 but not affected by 1C1 or 2C2 preincubation (Figs. 1*D* and S1*G*). Next, displacement experiments were performed in which BWSn cells were first preincubated with AF647-labelled VHH and then unlabeled VHH to determine their capacity to displace Siglec-1 bound labelled VHH. 1B5 and 1C1 could partially displace each other while 2C2 binding was unaffected (Figs. 1*E* and S1*H*). Conversely, 2C2 was not capable of displacing 1B5 or 1C1 (Figs. 1*E* and S1*H*). Taken together, these results imply that 1B5 and 1C1 have a similar epitope that does not overlap with 2C2, while the epitope of 2C2 overlaps with 7-239. In addition, the binding of 1B5 or 1C1 together with 7-239 appeared to be mutually enhancing.

Considering that mAb 7D2 blocks ligand binding and the binding of all three VHH was inhibited by 7D2, we hypothesized that the epitopes of all VHH are located on or near of Siglec-1-D1 (48). We therefore generated recombinant Siglec-1-D1 and tested binding of our VHH in an ELISA using recombinant Siglec-1 D1 or full length Siglec-1-D1-17. 2C2 and mAb 7-239 and 7D2 bound to both Siglec-1-D1 and Siglec-1-D1-17, indicating that they have epitopes in D1 (Fig. 1*F*). Interestingly, 1B5 and 1C1 did not bind to Siglec-1-D1, but only to Siglec-1-D1-17. Since 1B5 and 1C1 were also displaced by 7D2 that binds to D1, this suggested that they might have an epitope on the interface between Siglec-1-D1 and D2, although we can not formally exclude that the absence of D2 interferes with the conformation of D1.

Finally, we modeled the binding epitopes of our VHH using Alphafold3 (AF3) with either Siglec-1-D1 or Siglec-1-D1-2 as input. 2C2 was preferentially modeled on the ligand binding site of Siglec-1-D1 in a head-on orientation (49). The Siglec-1-D1/2C2 fold had a high predicted template modeling score (pTM) of 0.86 and high interface predicted template modeling (ipTM=0.8) score and overall confident predicted local distance difference test score (pLDDT>0.7) with the exception of CDR3 (Low (70 > pLDDT > 50)) (Fig. S1, *I* and *J*). 1B5 and 1C1 were modeled onto the interface between Siglec-1-D1 and 2, opposite the ligand binding domain, with a high modelling confidence for both 1B5 (pTM=0.86, ipTM=0.88) and 1C1 (pTM=0.80, ipTM=0.85) (Fig. S1, *I* and *J*). The Siglec-1-D1/2C2 fold was superimposed onto Siglec-1-D1-2/1B5 and 1C1 folds and is shown in Figure 1*G*. Interestingly, 2C2 was modeled onto R97 of Siglec-1-D1, which is known to be essential for ligand binding as mutation of this residue (R97A) abolishes Siglec-1 ligand interactions (50). In the AF3 model, the mutation R97A decreased output confidence significantly (pTM=0.27, ipTM=0.6). We therefore tested the binding of our VHH to HEK293T cells expressing either WT or R97A Siglec-1 by flow cytometry. VHH 1B5 and 1C1 and antibodies 7-239 and 7D2 could bind to both Siglec-1 forms, whereas 2C2 could only bind to WT Siglec-1 but not the R97A mutant, supporting the AF3 prediction (Figs. 1*H* and S1*K*). In conclusion, 2C2 binds specifically to the ligand binding site of Siglec-1, whereas 1B5 and 1C1 have predicted epitopes on the interface between D1 and D2.

### Siglec-1 binding VHH ortho- and allosterically modulate ligand binding

To address whether our VHHs can interfere with Siglec-1 ligand interactions, we preincubated Siglec-1 expressing cells with VHH or blocking mAb 7-239 (51), followed by incubation with ganglioside (GM3) containing DiD-labeled liposomes, which mimic Siglec-1/pathogen interactions (12, 30) (Figs. 2, *A* and *B* and S2*A*). 2C2 completely blocked liposome binding (IC_50_: 26.3 ± 2.5 nM), similar to mAb 7-239, and since it binds to the ligand binding site, it appears to be an orthosteric modulator (Figs. 2*B* and S2*B*). Unexpectedly, 1B5 and 1C1 enhanced GM3 liposome binding (Fig. 2*B*). To determine whether the effects of our VHHs on Siglec-1 ligand interactions was specific to GM3 or a more general effect, the Siglec-1-binding gangliosides GM1, GM3, GD1a, and GD3 liposomes were tested. Pre-incubation with 2C2 blocked all liposome tested regardless of the ligand, similar to 7-239 (Fig. 2*C*). 1B5 significantly enhanced ligand binding independent of the ganglioside tested, while 1C1 showed a similar, although nonsignificant, trend (Fig. 2*C*). In conclusion, our VHH either orthosterically (2C2) or allosterically (1B5/1C1) modulate ligand binding by either blocking or enhancing interactions between ganglioside-containing liposomes and Siglec-1.

**Figure 2:**
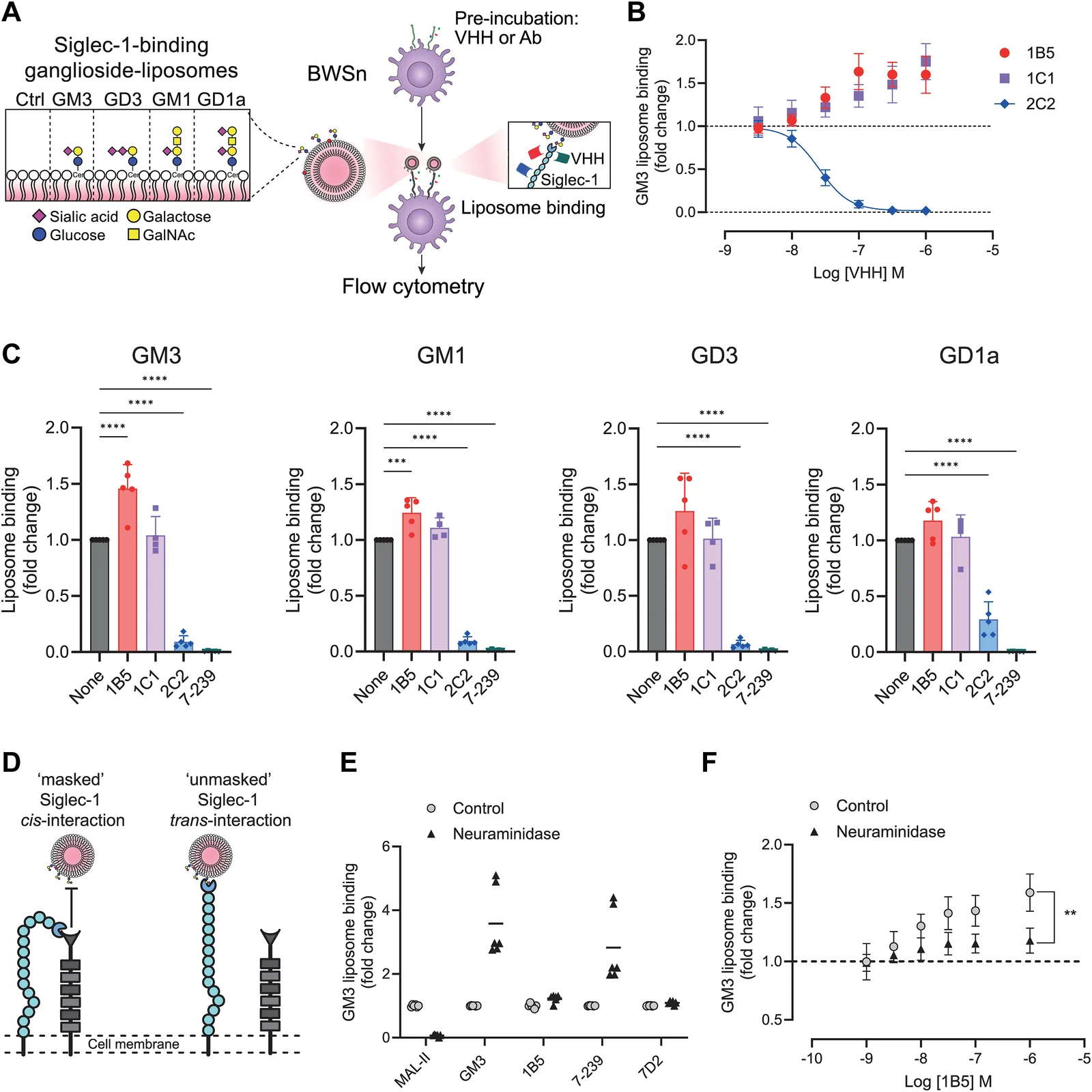
**Modulation of Siglec-1 ligand binding by VHH.***A*, schematic overview of experiments to determine effect of VHH or conventional antibody on ganglioside liposome binding. *B*, GM3 liposome binding after preincubation of BWSn cells with VHH. *C*, ganglioside liposomes binding to BWSn cells after preincubation with VHH or conventional antibody 7-239. *D*, cartoon of Siglec-1 *cis* and *trans* interactions. *E*, binding of lectin MAL-II, GM3 liposome, 1B5, conventional antibodies 7-239 and 7D2 to BWSn cells that were either treated with neuraminidase or a buffer control. *F*, GM3 liposome binding after preincubation of BWSn cells with 1B5 as detected by flow cytometry. Cells were treated prior to VHH binding with neuraminidase or a buffer control. Fold change data is shown of liposome DiD signal normalized to a no VHH control. Data is shown as mean ± *SD* of *n* = 3 to 5 experiments (*t* test:∗∗*p* < 0.01, one-way ANOVA: :∗∗∗*p* < 0.001, ∗∗∗∗*p* < 0.0001). Siglec-1, Sialic acid-binding immunoglobulin-like lectin 1.

### VHH 1B5 potentially modulates Siglec-1 *cis-*interactions

Siglec-1 is known to engage ligands in both *trans* and *cis-*interactions whereby *cis*-interactions ‘mask’ the receptor for binding to soluble ligands and ligands on other cells (Fig. 2*D*). Therefore, we speculated that the enhancement in liposome binding observed with 1B5 and 1C1 could partially be the result of the VHH modulating *cis*-interactions. To test this hypothesis, we pretreated BWSn cells with neuraminidase to remove cell surface sialic acid resulting in unmasking of Siglec-1 from *cis*-interactions. Successful desialylation of BWSn cells was confirmed by a significant reduction in binding of MAL-II, a plant lectin that specifically engages sialic acid in an (α-2,3) linkage on O-glycans exposed on the cell surface (Fig. 2*E*). Binding of GM3 liposomes and mAb 7-239 increased significantly when cells were treated with neuraminidase, but 1B5 and mAb 7D2 binding was only enhanced to a small extent (Fig. 2*E*). Next, cells treated with neuraminidase were preincubated with 1B5 and subsequently incubated with DiD-labeled GM3 liposomes. Overall, GM3 liposome binding was significantly enhanced by neuraminidase, but the 1B5 mediated effect was significantly reduced for the neuraminidase-treated cells when compared to the buffer only control (Fig. 2*F*). Taken together, these results suggest that the ligand binding enhancing effect of 1B5 could be mediated through modulation of Siglec-1 *cis-*interactions.

### VHH 2C2 blocks Siglec-1-mediated *C. jejuni* binding

Siglec-1 binds pathogens such as the gram-negative bacterium *C. jejuni* that expresses LOS structures with sialylated moieties mimicking Siglec-1-binding gangliosides on its outer membrane. To investigate the modulating effect of our VHHs on the binding of *C. jejuni* to Siglec-1 expressing cells, we incubated TSn with heat-inactivated fluorescein isothiocynanate (FITC)-labeled *C. jejuni* strains expressing various ganglioside mimics (GB14 = GM1a, GB23 = GM2, GB31 = GM1a/GD1a) (38). All three *C. jejuni* strains bound to TSn cells but not to the parental cell line THP-1 (Fig. S3, *A* and *B*). Dose titration curves demonstrated a blocking effect of 2C2 and an enhancing effect of 1B5 on *C. jejuni* binding for all three strains tested (Fig. 3*A*). Nonetheless, blocking of *C. jejuni* by 2C2 was less efficient than 2C2 blocking of the smaller ganglioside liposomes (Table S1). The binding of the *C. jejuni* strains to TSn cells was also visualized by fluorescent microscopy. A clear association with the cell membrane was observed in the untreated condition or in the presence of 1B5, whereas 2C2 and 7-239 blocked all three *C. jejuni* strains from binding TSn cells (Figs. 3*B* and S3*C*). Next we assessed the effects of the three VHH on binding of *C. jejuni* strains GB14, 23, and 31 by Siglec-1^+^ moDCs. Although moDCs express multiple Siglec receptors, binding of all strains by moDCs was Siglec-1 dependent as it could be blocked by preincubation with 7-239 similar to what was observed for macrophages (38) (Fig. 3*C*). Preincubation with 500 nM of 2C2 significantly blocked *C. jejuni* binding regardless of the strain, while 1B5 and 1C1 demonstrated an enhanced binding of all strains (Figs. 3*C* and S3*D*). All three *C. jejuni* strains tested induced production of IL-6, which could largely be abrogated by preincubation with 7-239 and 2C2, whereas VHH alone could not activate moDCs (Figs. 3*D* and S3*E*). In conclusion, these data reveal that our VHH 1B5 and 1C1 enhance the binding function of Siglec-1, while 2C2 is capable of blocking *C. jejuni* binding and subsequent activation of moDCs.

**Figure 3:**
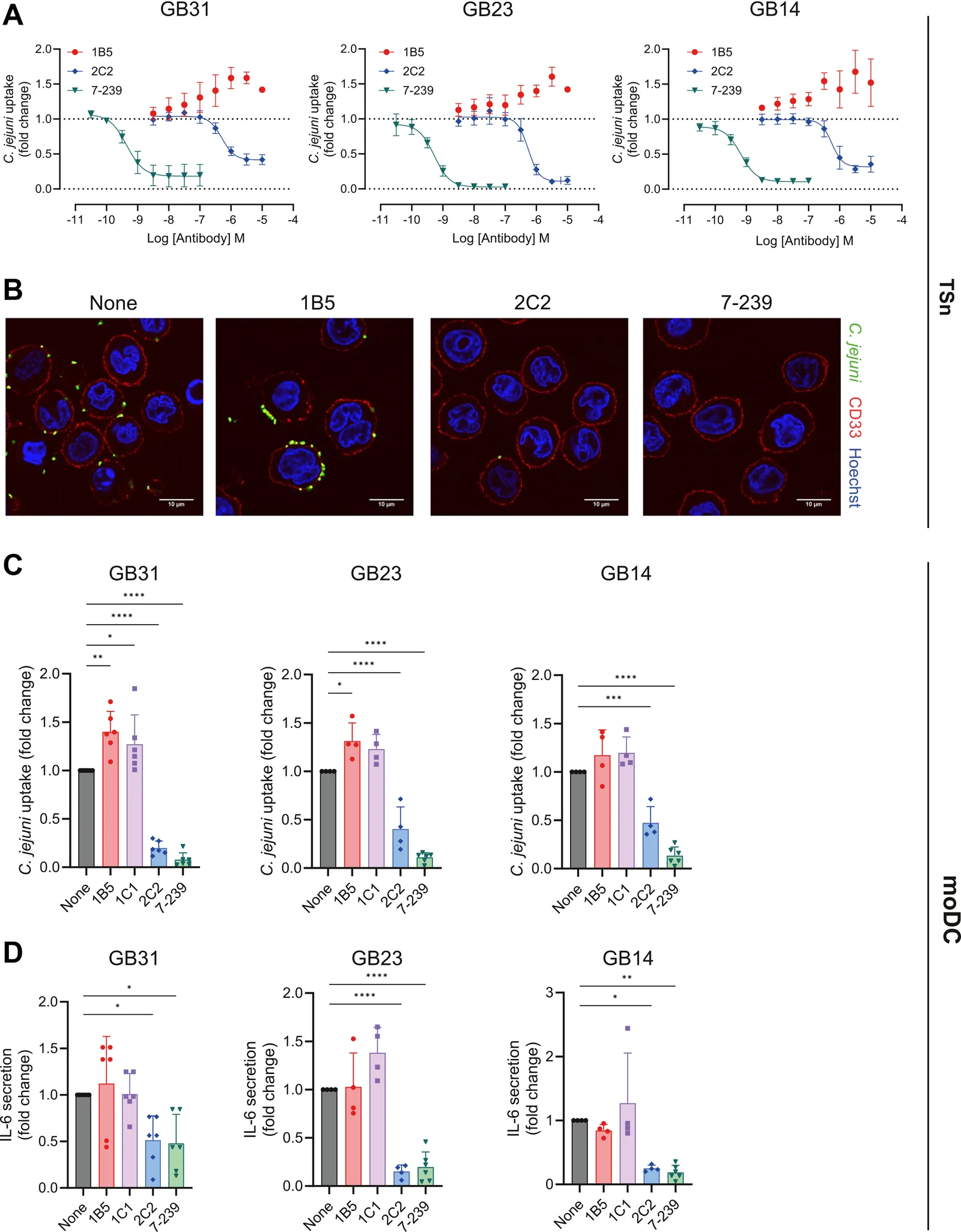
**VHH modulate *C. jejuni* binding to Siglec-1.***A*, binding data of FITC-labeled *C. jejuni* strains GB31, GB23, and GB14 to Siglec-1 expressing THP1 cells (TSn) as determined by flow cytometry. TSn cells were pre-incubated with VHH or conventional antibody 7-239 before incubation with *C. jejuni*. Fold change data is normalized to a no VHH/antibody control. Data is shown as mean ± *SEM* of *n* = 3 experiments. *B*, representative images of FITC-labelled GB31 *C. jejuni* binding to TSn cells preincubated with 7-239 or VHH. TSn membranes detected with anti-CD33-AF647 antibody. Nucleus is stained with Hoechst. *C*, binding of *C. jejuni* strains GB31, GB23, and GB14 by moDCs preincubated with VHH or conventional antibody as determined by flow cytometry. Normalized data is shown as fold changeover no VHH control of 4 to six donors (one way ANOVA: ∗∗*p* < 0.01, *∗∗∗p* < 0.001, *∗∗∗∗p* < 0.0001). *D*, IL-6 secretion by moDCs, preincubated with VHH or conventional antibody, followed by a 6 h incubation with *C. jejuni* strains GB31, GB23, and GB14 at 37 °C. IL-6 amounts were determined by ELISA. Normalized data is shown as fold changeover no VHH control, mean ± *SD* of 4 to six donors (one way ANOVA: ∗*p* < 0.05, *∗∗p* < 0.001, *∗∗∗∗p* < 0.0001). Siglec-1, Sialic acid-binding immunoglobulin-like lectin 1.

### Siglec-1-binding VHH bind to primary cells

Previously, we have shown that circulating immune cells in the blood, Axl^+^ Siglec-6^+^ DC cells (also known as Axl^+^ DC, pre-DC or transitional DC) express Siglec-1 constitutively and can bind ganglioside liposomes (51). To determine VHH binding to human Siglec-1^+^ primary cells, peripheral blood mononuclear cells (PBMCs) were isolated from healthy donors, and VHH binding was assessed *ex vivo*. Uniform Manifold Approximation and Projection dimensionality reduction analysis was performed on HLA-DR^+^ Lin^-^ DCs using markers for DC lineages: CD14, CD163, CD123, CD11c, CD1c, CD141, Axl, Siglec-6, and Siglec-1. Various DC subsets, including DC1, DC2, DC3, pDC, and Axl^+^ DC cells, were identified with conventional sequential gating strategy and overlaid on the Uniform Manifold Approximation and Projection (Figs. 4*A* and S4*A*). Siglec-1 was shown to be predominantly expressed by Axl^+^ DCs and 1B5, 1C1 and 2C2 were shown to bind to this cluster specifically (Fig. 4, *A* and *B*).

**Figure 4:**
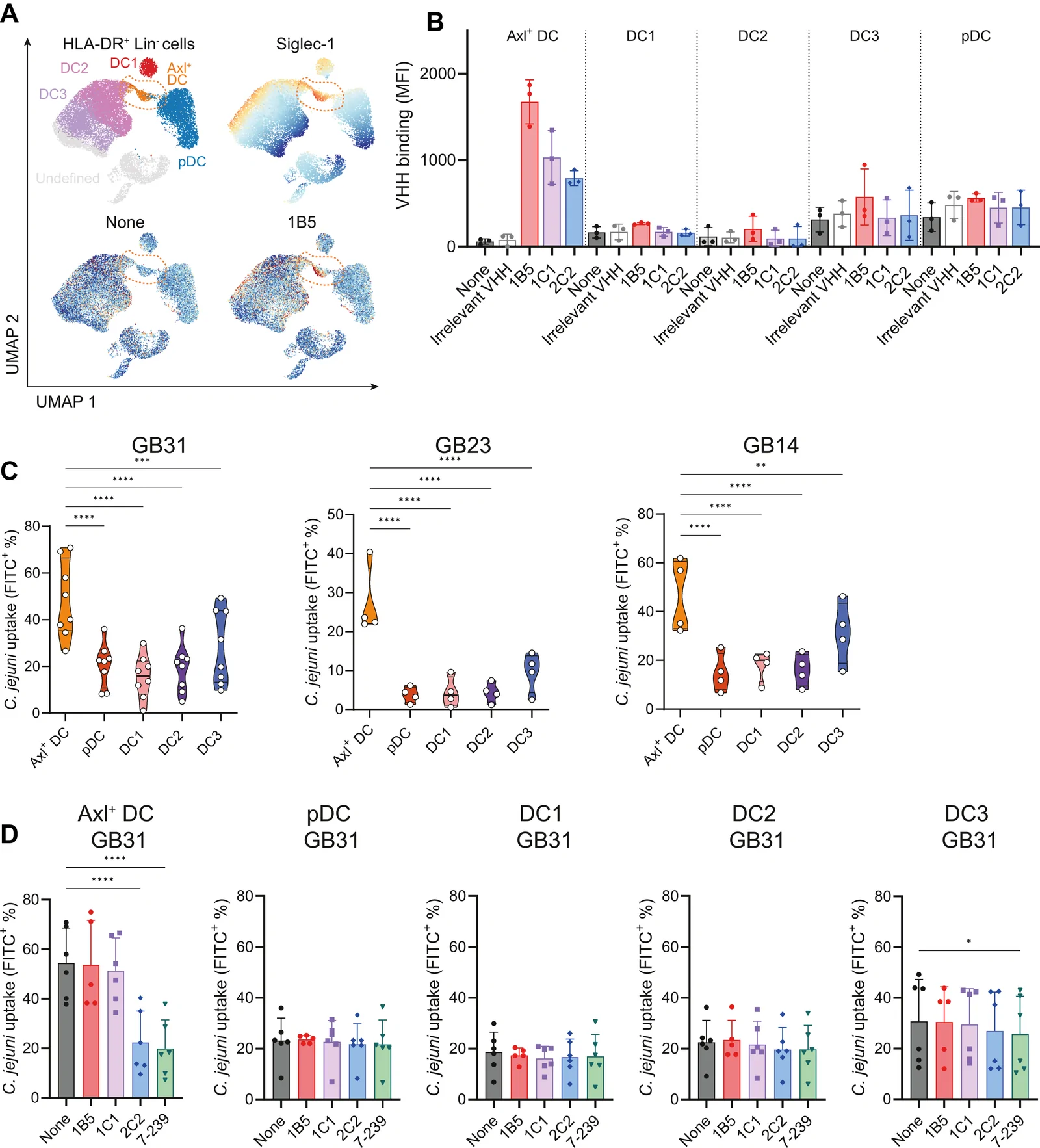
**VHH bind to Siglec-1-expressing DCs and 2C2 blocks *C. jejuni* uptake.***A*, high dimensionality reduction analysis by Uniform Manifold Approximation and Projection of HLA-DR^+^ Lin(CD3/CD19/CD56/CD88)^−^ DCs using markers: CD123, CD11c, CD1c, CD141, Axl, Siglec-6, and Siglec-1. Identification of DC subsets DC1, DC2, DC3, pDC, and Axl^+^ DC cells was done by conventional sequential gating. Binding of Siglec-1 specific VHH is restricted to cells expressing Siglec-1 on Uniform Manifold Approximation and Projection. *B*, binding of VHH to DCs as determined by flow cytometry. Data is shown as mean ± *SD* of at least three donors. *C*, percentages of FITC^+^ cells after incubation with FITC-labelled *C. jejuni* strains GB31, 23 and 14 as determined by flow cytometry. Data is shown as mean ± *SD* for 4 to eight donors (one way ANOVA: ∗∗*p* < 0.01, *∗∗∗p* < 0.001, *∗∗∗∗p* < 0.0001). *D*, percentages of FITC^+^ cells after preincubation with VHH or conventional antibody 7 to 239 followed by incubation with FITC-labelled *C. jejuni* strains GB31, GB23, & GB14 as determined by flow cytometry. Data is shown as mean ± *SD* for 5 to 6 donors (one way ANOVA: *∗∗∗∗p* < 0.0001). DC, dendritic cell; Siglec-1, Sialic acid-binding immunoglobulin-like lectin 1.

### Axl^+^ Siglec-6^+^ DC cells take up *C. jejuni*

Next, we assessed the binding of *C. jejuni* by primary APCs in the PBMC and the effect of 1B5, 1C1 and 2C2. Both Axl^+^ DCs and DC3 showed binding of all three strains of *C. jejuni* tested (Fig. 4*C*). Interestingly, Axl^+^ DC binding could be blocked by 7-239, but DC3 binding appeared not to be Siglec-1 dependent. In line with our previous data, 2C2 significantly blocked bacterial binding by Axl^+^ DCs, while clones 1B5 and 1C1 did not interfere with *C. jejuni* binding by Axl^+^ DCs (Fig. 4*D*). No effect of VHH was observed on the binding by DC3. Moderate levels of binding were observed by classical (CD14^+^, CD16^−^) and intermediate monocytes (CD14^+^, CD16^+^) of strains GB31 and GB14 and lower levels of binding of GB23 (Fig. S4*B*). Similar to DC3, binding by classical and intermediate monocytes was not mediated by Siglec-1 (Fig. S4, *C–E*). In summary, we have shown that our VHH bind to primary DCs expressing Siglec-1 and that 2C2 specifically blocks *C. jejuni* binding to Siglec-1.

### VHH 2C2 blocks Siglec-1 mediated SARS-CoV-2 binding

Siglec-1 is known to capture viruses such as SARS-CoV-2 *via* binding to sialylated gangliosides or glycoproteins present in the viral envelope (22, 29). Here we tested binding of WT SARS-CoV2 recombinant spike protein (S1+S2 trimer) to cells. BWSn cells were pre-incubated with either 500 nM VHH or 100 nM antibody prior to incubation with recombinant spike protein (Figs. 5*A* and S5, *A* and *B*). 1B5 and 1C1 did not affect spike protein binding to Siglec-1-expressing cells whereas 2C2 blocked binding to the same degree as mAb 7-239 (Figs. 5*A* and S5, *A* and *B*). To investigate whether the VHH could block Siglec-1-mediated binding of SARS-CoV-2, TSn and moDC cells were preincubated with 500 nM of VHH or 100 nM of mAb 7-239 before incubation with wild-type SARS-CoV-2 (hCoV-19/Italy). Viral binding was assessed by the presence of SARS-CoV-2 *orf1b* RNA in cell lysates (Fig. 5, *B* and *C*). While significant amounts of virus binding were detected with 1B5 and 1C1, 2C2 completely blocked SARS-Cov-2 binding to TSn cells (Fig. 5*B*). Similarly, 1B5 marginally affected SARS-Cov-2 binding to moDCs, 2C2 significantly suppressed it, comparable to 7-239 (Fig. 5*C*). In contrast to TSn cells, moDC also express DC-SIGN a known co-receptor for SARS-CoV-2, which could mediate virus binding when Siglec-1 binding is inhibited, explaining the higher background (Fig. 5*C*) (25). Taken together, we have shown that 2C2 blocks SARS-Cov-2 binding to Siglec-1.

**Figure 5:**
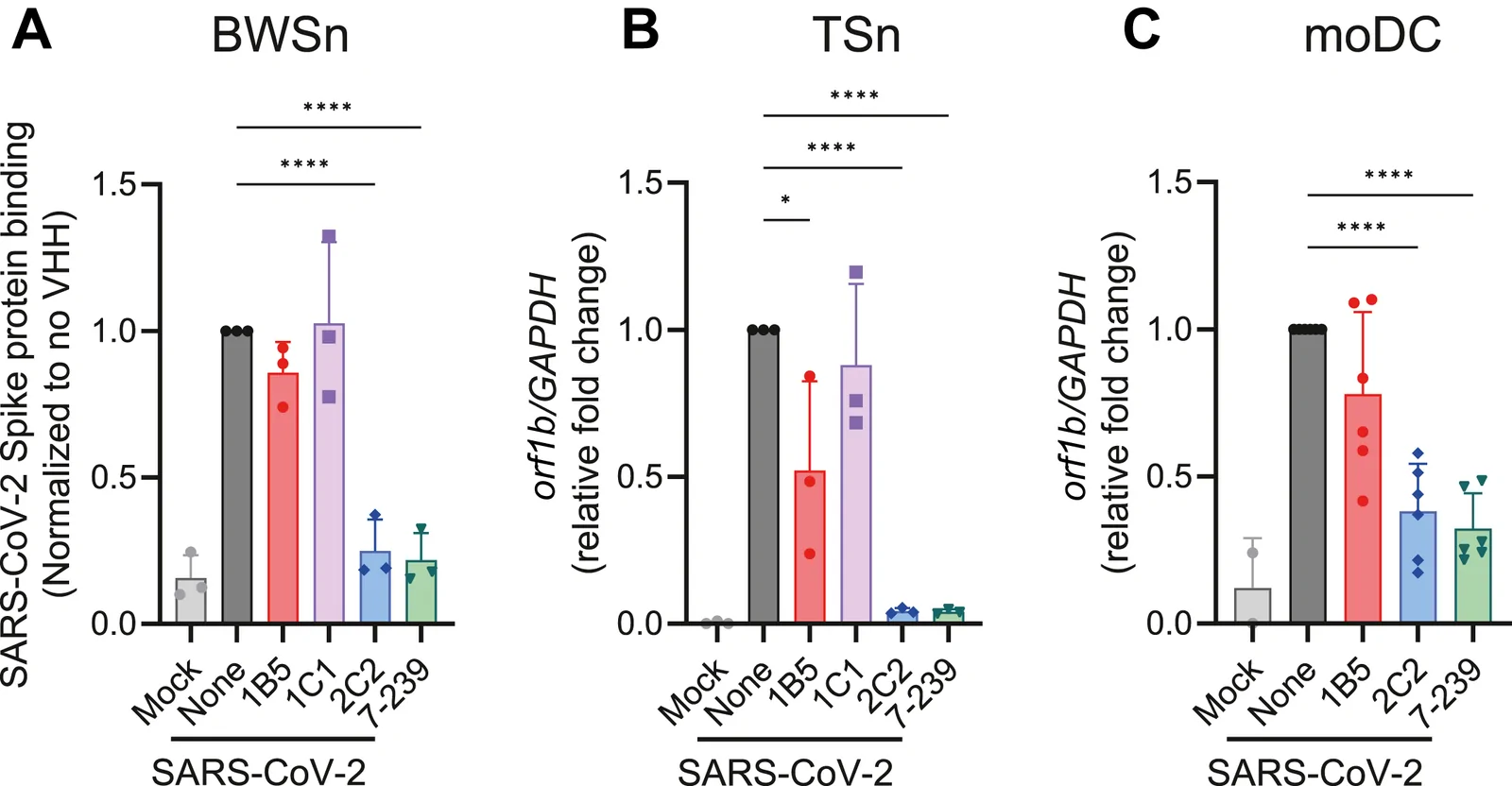
**Siglec-1-specific VHH 2C2 blocks SARS-CoV-2 binding.***A*, recombinant spike protein binding to BWSn cells after VHH/antibody preincubation as detected by flow cytometry. Fold change data is shown as mean ± *SD* for *n* = 3 experiments, normalized to no VHH condition (one way ANOVA: *∗∗∗∗p* < 0.0001). *B*, qPCR of SARS-CoV-2 *orf1b* from cell lysates of Siglec-1 expressing THP-1 cells (TSn) or *C*) moDCs. Data is normalized to GAPDH. *B*, data is shown as mean ± *SD* for *n* = 3 experiments. *C*, data is shown as mean ± *SD* for six donors (one way ANOVA: *∗p* < 0.05, *∗∗∗∗p* < 0.0001). Siglec-1, Sialic acid-binding immunoglobulin-like lectin 1.

## Discussion

Here we report VHH that allosterically enhance or orthosterically block ligand binding to Siglec-1. Remarkably, we show that 1B5 and 1C1 increase ligand binding to Siglec-1 on moDCs. In contrast, 2C2 effectively blocks Siglec-1 ligand interactions and 2C2-mediated inhibition of sialylated pathogen binding such as *C. jejuni* and SARS-Cov-2 by moDCs, resulted in decreased inflammatory cytokine production by APCs. Furthermore, we reveal for the first time that Axl^+^ DCs can bind *C. jejuni* in a Siglec-1-dependent manner, which was also blocked by 2C2. These results highlight the potential of VHH as effective modulators of pathogen binding to Siglec-1 on primary cells.

To illuminate how our VHH differentially affect Siglec-1 interactions, we generated AF3 models of 1B5, 1C1 and 2C2 binding to the first two domains of Siglec-1 and confirmed the results with epitope mapping experiments for 2C2. Interestingly, AF3 docked 2C2 directly onto the ligand binding site in a head-on orientation in which R97 is inserted between CDR1 and CDR3 of 2C2. R97 is crucial for sialic acid binding to Siglec-1, as R97 forms a salt bridge with the carboxylate of sialic acid and thus, the mutant receptor (R97A) no longer binds sialylated ligands (52). Binding of 2C2 to D1 of the receptor was confirmed with recombinant Siglec-1-D1. Moreover, a single point mutation, R97A, in Siglec-1 was sufficient to abrogate 2C2 binding, identifying the ligand binding site as the interaction interface thus elucidating 2C2’s capacity to block ligand interactions.

AF3 was unable to confidently dock 1B5 and 1C1 onto Siglec-1-D1. Introduction of Siglec-1-D2 was sufficient to generate models with a high confidence. In the AF3 model, D1 is connected to D2 by a short linker. The interdomain interface is further stabilized by a predicted disulfide bridge between C17 of D1 with C147 of the D2. The AF3 models suggest that 1B5 and 1C1 target a binding site on the interface of D1-2, opposite the ligand binding site. These findings are supported by binding data using recombinant protein and antibody displacement experiments as both VHH required D1 and D2 for binding, but were blocked by 7D2, which exclusively binds to D1. Nonetheless, we cannot rule out 1B5 or 1C1 binding to solely D2.

The question remains how binding of 1B5 and 1C1 enhance ligand binding despite not interacting directly with the ligand binding site. VHH can act as conformational stabilizers locking target proteins in distinct conformations such as inactive or active states of GPCRs, or allosterically regulating enzyme activity (45, 53). In the case of Siglec-7 it has been shown that the CC’ loop in the Ig-like V-set domain has an ‘open’ form in the native state that undergoes a large conformational change, to a ‘closed’ orientation, upon binding of the ligand GT1b (54). However, for Siglec-1 no large scale conformational changes have been observed upon ligand binding (52, 55). Alternatively, due to the high levels of sialic acid found on the cell surface Siglecs can bind ligands on the same membrane in so called *cis*-interactions, which need to be unmasked for the receptor to engage in *trans*-interactions with ligands on opposing cells (10). Siglec-1 is the largest member of the CD33-related Siglec family with 17 extracellular Ig domains, which in the literature has been suggested to enable the receptor to escape inhibitory *cis-*interactions and predominantly engage ligands in *trans* (10). Nonetheless, it has been shown that soluble Siglec-1 ligand binding can be enhanced by de-sialylation of various cell types expressing Siglec-1, thereby unmasking the receptor (14, 15, 16, 56). Here, a significant increase in GM3 liposome and 7-239 antibody binding was observed upon neuraminidase treatment of Siglec-1 expressing BWSn cells, which indicates that Siglec-1 is engaged in *cis*-interactions on these cells. Neuraminidase treatment prior to VHH binding significantly reduced the enhancing effect of VHH 1B5 on GM3 liposome binding. We speculate that Siglec-1 is engaged in *cis*-interactions which can be unmasked by 1B5 binding resulting in enhanced GM3 liposome binding. In the neuraminidase treated condition, Siglec-1 is already unmasked and therefore no additional effect of 1B5 binding is seen on GM3 liposome binding. Alternatively, 1B5 binding to Siglec-1 could potentially affect the overall rigidity of the receptor making it less capable of engaging in *cis*-interactions and thereby increasing soluble ligand binding. The degree of *cis*-interactions may vary between various Siglec-1^+^ cells and may potentially influence the effect of 1B5 on ligand binding. While we observed strong enhancing effects of 1B5 and 1C1 on BWSn and TSn cells, this was not observed with 1B5 binding to Axl^+^ DCs and further studies will be necessary to determine whether Axl^+^ DCs have less *cis-*interacting Siglec-1.

Here, we investigated the effects of 1B5, 1C1, and 2C2 on Siglec-1 ligand binding in the context of bacterial and viral pathogens. Pathogens are known to incorporate host-like molecules to circumvent or manipulate the host immune response in a process called molecular mimicry (35, 57). In the case of *C. jejuni* infections, molecular mimicry is thought to be the cause of GBS, a deadly post-infectious neuropathy, resulting from *C. jejuni* antibodies cross-reacting with gangliosides on host peripheral nerves (35, 57). Sialylated LOS structures are known to enhance endocytosis of *C. jejuni* and subsequent translocation across intestinal epithelial cells (58, 59), but also mediate binding to Siglec-1 and uptake by macrophages and mouse DCs, resulting in enhanced cytokine production (23, 38, 39, 40). In line with this, we report *in vitro* binding of GBS-related strains GB14, GB23, and GB31 by TSn and moDCs in a Siglec-1-dependent manner, which could be either enhanced by 1B5/1C1 or blocked by 2C2 resulting in reduced IL-6 levels by moDCs. In a murine infection model, sialylated *C. jejuni* was shown to induce the production of GBS-associated cross-reactive antibodies targeting gangliosides and blocking murine Siglec-1 was sufficient to prevent the production of cross-reactive antibodies in this model (41). We show for the first time that Siglec-1^+^ Axl^+^ DCs but no other DC subsets bind to GBS-associated *C. jejuni* strains in a Siglec-1-dependent manner and that 2C2 specifically blocks this interaction. While a potential role for Axl^+^ DCs in the development of GBS is not known, further research into the therapeutic application of Siglec-1 blocking is warranted.

Multiple enveloped viruses, including SARS-Cov-2, are known to contain sialylated proteins and gangliosides and exploit binding to Siglec-1 to infect cells (22, 25, 26, 27, 30, 60). SARS-CoV-2 attachment to Siglec-1 on dendritic cells leads to trans-infection of other permissive cells, contributing to viral dissemination (25, 31). Instead of using neutralizing antibodies for each specific virus strain, blocking antibodies to Siglec-1 have been developed as a pan-antiviral therapy (61). Here we show that SARS-Cov-2 binding to TSn and moDCs was efficiently blocked by 2C2. Since VHH are smaller than antibodies and penetrate tissues better, Siglec-1 blocking VHH could be used as an alternative option for therapeutic pan-antiviral applications.

In this study, we have identified Siglec-1 binding VHH that either allosterically enhance or orthosterically block ligand binding. We show that these VHH modulate Siglec-1 interactions within the context of sialylated liposomes and pathogens (*C. jejuni* and SARS-CoV-2) thereby enabling the further study of Siglec-1’s role within infectious diseases. Finally, the identified Siglec-1-specific VHH are promising modalities for further investigation as potential therapeutics, as VHH can easily be engineered to generate multivalent proteins with enhanced affinities, or be made bi-specific to block multiple cross-pathogen receptors.

## Experimental procedures

### Cell culture

BW5147 cells overexpressing human Siglec-1 (BWSn), THP-1 cells overexpressing human Siglec-1 (TSn), parental THP-1 and BW5147 (BW; (47)) cells were maintained in RPMI 1640 (Thermo Fisher Scientific) complete medium, containing 10% fetal calf serum (Biowest), 50 U/ml penicillin, 50 μg/ml streptomycin and 2 mM glutamine (all from Thermo Fisher Scientific) at 37 °C and 5% CO_2_.

### Generation of monocyte-derived DCs (moDCs)

Monocytes were isolated using a Percoll gradient or CD14-magnetic beads (Miltenyi Biotec) and then were cultured for five to six days in RPMI 1640 complete medium (Thermo Fisher Scientific) containing 10% fetal calf serum (Biowest), 50 U/ml penicillin, 50 μg/ml streptomycin, and 2 mM glutamine (all from Thermo Fisher Scientific), in the presence of recombinant human IL-4 (500 U/ml) and GM-CSF (800 U/ml; both from Immunotools) at 37 °C and 5% CO_2_. For the final 48 h, moDCs were treated with recombinant human IFNα (100 U/ml, Immunotools).

### Generation of Siglec-1-targeting VHHs

VHHs were generated against human Siglec-1 by immunizing two llamas with cells and recombinant protein in four rounds. The first two rounds consisted of human moDCs, treated with IFNα to upregulate Siglec-1 expression, one round of THP-1 cells expressing Siglec-1 (TSn) (62), and a final round with recombinant human Siglec-1 (Ser20-Gln1641) (R&D Systems, 5197-SL). Peripheral blood was obtained from each llama and PBMCs were isolated after which RNA was isolated and reverse transcribed into cDNA. Genes of the variable domain of heavy chain only antibodies were amplified and ligated into the phagemid vector pPQ81 (QVQ) for generation of phage-display libraries using *E. coli* TG1 cells as previously described (63). Phage-display selections were performed using 5 μg/ml recombinant human Siglec-1 (Ser20-Gln1641) (R&D Systems, 5197-SL) coated in Maxisorp plates. Blocking was performed with 4% (w/v) skimmed milk in PBS and counter selections were performed using plates coated with human IgG1. Output phages were generated after either chemical or antibody (anti- Siglec-1, clone 7-239, (47)) elution. Afterwards, TG1 cells were infected by output phages and used to generate four 96 well masterplates containing periplasmic extracts which were then screened for binding to recombinant human Siglec-1. A total of 368 clones were screened for binding and subsequently sequenced resulting in 81 family clusters (CDR-HR homology >80%) of which 1 to 2 clones were selected for VHH production as previously described (63) and further analysis.

### VHH production

VHH sequences of selected clones were ordered from TWIST Bioscience with an N-terminal PelB sequence and C-terminal Myc and 6xhis tag and subcloned into pET28a+. VHH clones were transformed into LOBSTR-BL21(DE3)-RIL cells (Kerafast, EC1002). Starter cultures were grown in 2 ml Luria’s Broth medium supplemented with 50 μg/ml kanamycin and 2% glucose O/N at 37 °C, shaking at 280 rpm. Next, VHH were expressed by innoculating 500 ml autoinduction medium consisting of terrific broth base including trace elements (Foremedium, AIMTB2010) supplemented with 50 μg/ml kanamycin and grown for 56 h at 18 °C, shaking at 220 rpm. Cultures were harvested by centrifugation at 4000 rcf for 60 min at 4 °C. Cell pellets were incubated O/N at −20 °C after which they were resuspended in PBS supplemented with 1 mM MgCl_2_ and 5 μg/ml DNase-I and incubated on a rotor O/N at 4 °C. Lysates were clarified by centrifugation at 4000 rcf for 60 min at 4 °C and subsequently filtered through a 0.2 μm filter and supplemented with 30 mM imidazole. VHHs were purified by affinity chromatography using 5 ml His-Trap HP columns (Cytiva) and afterward imidazole was removed by gel filtration with a HiPrep 26/10 column (Cytiva).

### VHH conjugation with Alexa Fluor 647

VHH’s were expressed and purified with a C-terminal 6xhis-tag separated by a rigid-linker (SPSTPPTPSPSTPP) from a cysteine (AAK**C**KAA) used for maleimide conjugation (Massa et al., 2014). The VHH were subsequently reduced by adding 50 mM TCEP (Sigma-Aldrich) at a molar ratio of TCEP:VHH of 3:1 for 2 h at RT. Next, TCEP was removed by buffer exchange using a PD10 desalting column and Alexa Fluor 647 C_2_ Maleimide dissolved in DMSO was added to the VHH and incubated O/N at 4 °C on a roller in the dark. Next, free dye was removed with a PD10 desalting column. VHH were then concentrated using Vivaspin tubes (Sigma Aaldrich, Z629464) with a molecular weight cut-off of 3 kDa.

### Human Siglec-1 ELISA

His-tagged recombinant human Siglec-1 (Ser20-Gln1641) (R&D Systems, 5197-SL) or Siglec-1 domain 1 (Ser20-Thr136) were coated with a concentration of 5 μg/ml in coating buffer (0,2 M NaHCO_3_, pH=9,7) in Nunc MaxiSorp ELISA plates (Thermo Fisher Scientific) O/N at 4 °C. Plates were subsequently blocked with carbo free blocking buffer (Vector labs, SP-5040-125) for 30 min at 37 °C. Next, VHH and antibody incubations were performed in carbo free blocking buffer for 1 h at RT. VHH were detected with anti-his horseradish peroxide (HRP) conjugated antibodies (Biolegend). Anti-Siglec-1 antibodies with anti-mouse-HRP (Agilent, P0260). After each step wells were washed with PBS supplemented with Tween-20 (0.05% w/v). ELISA was developed by the addition of TMB substrate (Thermo Fisher Scientific, N301), the reaction was stopped by the addition of 0.3 M H_2_SO_4_ and absorbance was measured at 450 nm using a microplate spectrophotometer.

### VHH and antibody binding to cells

Cells were first preincubated with human Fc Block (BD Biosciences), True-Stain Monocyte Blocker (Biolegend), and viability dyes (Fixable viability dye eFluor 780, eBioscience, or Live Dead Blue, Life Technologies) in PBS at 4 °C for 10 min. For cells expressing Siglec-1-halotag, cells were stained with cell permeable halotag ligand (CA-MaP555, Tenova Pharmaceuticals) on ice for 30 min followed by five washing steps with PBS supplemented with BSA (0.5% w/v) (PBA). Next, cells were incubated at 4 °C with either VHH or antibodies for 30 min at 4 °C followed by washing with PBA after each antibody incubation step. VHH were detected with anti-Myc-tag antibody (Cell Signaling) or anti-his tag-AF647 antibody (Biolegend). In the epitope binning experiments VHH were labelled with AF647 dyes. Cells were fixed with 2% paraformaldehyde (PFA) for 10 min at 4 °C. Cells were stored at 4 °C up to 1 week until acquisition on Attune (Life Technologies), BD LSR Fortessa (BD Bioscience), or Aurora spectral flow cytometer (Cytek) and analyzed on FlowJo software (https://www.flowjo.com/flowjo/overview, Tree Star, Inc) or OMIQ (Dotmatics). In the case of pLent6.3-Siglec-1-halotag transfected HEK293T cells, successfully transfected cells were identified by halotag ligand Ca-MaP555 positive signal. VHH binding curves were analyzed with GraphPad Prism 10.6 and fitted with four parameter dose-response curve.

### Alpha Fold VHH binding prediction

Alpha Fold3 was used to predict VHH binding to human Siglec-1. The input sequence of human Siglec-1 (Uniprot Q9BZZ2) consisted of the first domain (Ser20-Glu138) for modeling interactions with VHH clone 2C2 and the first two domains (Ser20-233Gln) for modeling interactions with VHH clones 1B5 and 1C1. The model shown in the main text consists of the Siclec-1-D1/2C2 fold superimposed on the folds of Siglec-1-D1-2/1B5 or 1C1. Models were analyzed using UCSF Chimera-X (64). Coloring of the structures according to the pLDDT scores was done according to the color key shown in the figures with color gradient thresholds set at 50, 70 and 90.

### Production of recombinant human Siglec-1 domain 1

Siglec-1 gene fragment encoding Siclec-1-D1 (Ser20-Glu138) were cloned into pFastBacNKI-GP67-LIC-3C-2xStrepII-10xhis, using ligation independent cloning (65). Proteins are expressed with an N-terminal GP67 signal peptide and a C-terminal TwinstrepII-10xhis tag. Plasmids were transformed into EMBACY cells and bacmid DNA was isolated from three individual positive (white) colonies, according to the Bac-to-Bac manual (Thermo Fisher Scientific). Sf9 insect cells were plated in 6-well plates and the cells were transfected with bacmid DNA. After three days at 28 °C, the medium containing secreted virus particles was harvested (P0 virus) and added to 25 ml of sf9 suspension cultures. After three days, cells were monitored for growth, cell diameter and YFP expression to confirm virus production. Cells were removed by centrifugation (4 min at 900*g*) and medium was collected (P1 virus stock).

For protein expression, sf9 cells were infected with P1 virus (1 ml P1 virus added to 500 ml 1 × 10^6^ cells/ml sf9 cells). After three days, cells were removed by centrifugation (15 min at 900*g*) and medium was collected. The pH of the medium was adjusted to pH 8.0 by addition of 50 ml Tris-HCl pH 8.0 to 500 ml solution and precipitates were removed by centrifugation (10 min at 8000*g*). The solution was passed over 1.5 ml of Nickel Indigo beads (Cube Biotech). Beads were washed with 15 ml 25 mM Hepes pH 7.5, 200 mM NacL, 20 mM imidazole and protein was eluted using the same buffer including 300 mM imidazole. Elution fractions were pooled and passed over 1 ml of Streptactin XT beads (IBA Lifesciences). Beads were washed with 25 mM Hepes pH 7.5, 200 mM NacL before Siglec-1 proteins were eluted in the same buffer, supplemented with 50 mM biotin. Purified proteins were concentrated in Vivaspin concentrators (VIVAproducts) and dialyzed against 1 L of 25 mM Hepes pH 7.5, 200 mM Nacl. Protein aliquots were snap frozen in liquid nitrogen and stored at −80 °C.

### Surface plasmon resonance binding experiments

The binding affinities of VHHs to Siglec-1 were determined using surface plasmon resonance on a Biacore 1S+ instrument (Cytiva Life Sciences). Siglec-1-FL was immobilized on a CM5 sensor chip (Cytiva Life Sciences) *via* amine coupling in 10 mM glycine buffer, pH 5.0. Serial twofold dilutions of VHHs were injected over the chip surface in HBS-P+ buffer (10 mM Hepes, pH 7.4; 150 mM NaCl; 0.05% v/v Surfactant P20; Cytiva Life Sciences) using a single-cycle injection procedure. Binding responses were processed by double referencing, involving subtraction of both the reference surface and buffer blank responses. Data were analyzed using Biacore Insight Software (https://www.cytivalifesciences.com/en/us/products/items/biacore-insight-evaluation-software-p-23528?psmenu=2, Cytiva Life Sciences). Binding data for 1B5 and 2C2 were analyzed using a 1:1 kinetic binding model fit. Due to its very fast dissociation rate, which approached the experimental detection limit, the binding data for 1C1 were analyzed using an equilibrium binding model. Figures were prepared using GraphPad Prism (https://www.graphpad.com, GraphPad Software, LLC).

### Construction of Siglec-1-halotag expression vector

Siglec-1 WT and R97A (R116A when signal peptide is included) receptor mutant were designed with a c-terminal halotag separated by a small flexible linker (GGGGSGGGGS). Siglec-1 DNA inserts were ordered from Twist Bioscience in two parts: part-1 (Siglec-1: 1-3371 bp) had a 5′ BamHI and 3′ XbaI restriction site, part-2 (Siglec-1: 3372-5127 bp + halotag) had a 5′ XbaI and 3′ BstBI restriction site. A pLenti-6.3 expression vector with a CMV promoter transcribing a 5′ BamHI and 3′ XbaI flanked irrelevant gene with downstream 3′ BstBI restriction site. The irrelevant gene was digested with BamHI and XbaI (FastDigest, Thermo Fisher) before digestion and ligation part-1 into the vector. Next, the pLenti-6.3-Siglec-1-Part-1 vector was digested with XbaI and BstBI and Siglec-1-halotag was digested and ligated into the vector to generate a pLenti-6.3-Siglec-1-halotag vector containing WT or R97A mutant Siglec-1. Vector construction was verified by wholeplasmid sequencing (Eurofins).

### Siglec-1-halotag expression

HEK293T cells were seeded in T75 flasks at three million cells per flask in DMEM (10% FCS, 1% penicillin and streptomycin) and incubated O/N at 37 °C and 5% CO_2_. The next day medium was refreshed and cells were transfected with 10 μg receptor DNA using the PEI method in a 1:6 ratio of DNA to PEI (Polysciences Inc) and incubated O/N at 37 °C and 5% CO_2_. The following day medium was refreshed and cells were incubated another 48 h at 37 °C and 5% CO_2_. Next, cells were collected by scraping and used for flow cytometry analysis as described above.

### *C. jejuni* fluorescein isothiocynanate labelling

In this study GBS associated *C. jejuni* strains GB14 (ganglioside mimic GM1a, LOS class C), GB23 (ganglioside mimic GM2, LOS class A), and GB31 (ganglioside mimic GD1a, GM1a, LOS class A) were used (previously described by (23)). The *C. jejuni* strains originate from a GBS–related strain collection of the Erasmus MC, Rotterdam, and the Netherlands. Freshly cultured *C. jejuni* were harvested in PBS and incubated with fluorescein isothiocynanate (FITC) with shaking for 60 min at RT in the dark. Bacteria were then washed three times in PBS before being resuspended in PBS supplemented with 2 mM MgCl_2_. Afterwards, *C. jejuni* was heat inactivated by 45 min incubation at 56 °C in the dark and subsequently washed three times with PBS after which bacterial solutions were transferred to storage medium (PBS + glycerol) and stored at −80 °C. Thawed bacteria were washed two times in PBS before being used in binding experiments.

### Liposome binding

Siglec-1-targeting liposomes were generated as previously described (48). Liposome binding experiments were performed with BWSn cells. Cells were preincubated with VHH or conventional antibodies as indicated before being incubated with ganglioside-liposomes (100 μM) for 30 min at 4 °C. In the case of neuraminidase treatment, cells were washed three times with PBS prior to incubation with 0.4 U/ml of Neuraminidase from *Clostridium perfringens* (Roche, Cat. No.: 11585886001) or as control PBS, for 1 h at 37 °C, shaking at 700 rpm. Cells were subsequently washed three times with carbo free blocking buffer (Vector Labs, Cat. No.: SP-5040-125) before VHH/liposome binding. De-sialylation was evaluated by loss of biotinylated Maackia Amurensis Lectin II (MAL II) (Vector Labs, Cat. No.: B-1265-1) binding. Cells were then fixed and acquired on flow cytometry. Liposome binding curves were fitted with log(inhibitor) *versus* response -Variable slope in GraphPad Prism 10.6 to determine VHH IC_50_ values.

### *C. jejuni* binding

A cell to bacteria ratio of 1:10 or 1:100 was used in binding experiments. Cells were preincubated with VHH or antibodies where indicated for 30 min at 4 °C before being incubated with FITC labelled *C. jejuni* for 2 h at 37 °C for binding. After which cells were washed once with RPMI supplemented with 1% FCS and then stained for 10 min with Fixable viability dye eFluor 780 (eBioscience) for 10 min at 4 °C. Cells were then washed with PBA before being fixed with 2% PFA for 10 min in the dark at 4 °C. Binding was then measured by flow cytometry. In experiments where cytokine secretion was measured, moDCs were preincubated with VHH and antibodies before a six hour incubation with FITC labelled *C. jejuni*. Next, cells were pelleted by centrifugation and the supernatant was subjected to an IL-6 ELISA (Biolegend, Cat. No.: 430501). In the experiments were PBMCs were required they were isolated from heparinized blood by density gradient centrifugation (Lymphoprep; Axis-Shield PoC AS). Following PBS washes, cells were further processed for *C. jejuni* binding and flow cytometry, as described above. *C. jejuni* binding curves were fitted with log(inhibitor) versus response -Variable slope in GraphPad Prism 10.6 to determine VHH IC_50_ values.

### SARS-CoV-2 spike probe generation

Recombinant SARS-CoV-2 spike protein was produced as previously described (66). Afterwards, the recombinant SARS-CoV-2 spike protein was biotinylated using a BirA500551 biotin-ligase reaction kit (Avidity). Next, AF647-conjugated streptavidin was used to multerimize the biotinylated recombinant spike protein in a 2:1 M ratio at 4 °C for 1 h and free binding sites were blocked by adding 10 μM D-biotin for 30 min at 4 °C as described before (67).

### SARS-CoV-2 recombinant spike protein binding

BWSn cells were first stained with CellTrace violet (Thermo Fisher Scientific) for 7 min at 37 °C followed by a wash with RPMI 1640 (Thermo Fisher Scientific) complete medium, containing 10% fetal calf serum (Biowest), 50 U/ml penicillin, 50 μg/ml streptomycin and 2 mM glutamine (all from Thermo Fisher Scientific). Next, BW5147 and BWSn cells were stained for 10 min with fixable viability dye eFluor 520 (eBioscience) at 4 °C. Cells were then incubated with VHH or 7 to 239 antibody for 30 min at 4 °C. Next, recombinant AF647-labelled multimerized SARS-CoV-2 spike protein was added and incubated with cells for 30 min at 4 °C. Cells were then washed two times with PBA followed by fixation with 2% PFA for 20 min. Cells were then analyzed by flow cytometry.

### SARS-CoV-2 virus production

The following reagent was obtained from Dr Maria R. Capobianchi through BEI Resources, NIAID, NIH: SARS-related coronavirus 2, Isolate Italy-INMI1, NR-52284, originally isolated in January 2020 in Rome, Italy. VeroE6 cells (ATCC CRL-1586) were inoculated with the SARS-CoV-2 isolate and used for reproduction of virus stocks, as described previously (68).

### SARS-CoV-2 virus binding

THP-1 and TSn cell or moDCs were incubated with the SARS-CoV-2 isolate (hCoV-19/Italy; TCID50 10^4^ for 4 h at 37 °C. After 4 h cells were washed extensively to remove unbound virus. Cells were lysed and cellular and viral RNA was isolated using the QIAamp Viral RNA Mini Kit (Qiagen) according to the manufacturer’s protocol. cDNA was synthesized with the M-MLV reverse-transcriptase kit (Promega) and diluted 1:5 before further application. PCR amplification was performed in the presence of SYBR green (Thermo Fisher Scientific) in a 7500 Fast Realtime PCR System (Applied Biosystems). The normalized amount of target mRNA was calculated from the Ct values obtained for both target and household mRNA with the equation Nt = 2Ct ^(GAPDH)-Ct(target)^. The following primers were used: GAPDH, forward primer (CCATGTTCGTCATGGGTGTG), reverse primer (GGTGCTAAGCAGTTGGTGGTG). *orf1b*, forward primer (TGGGGTTTTACAGGTAACCT), reverse primer (AACACGCTTAACAAAGCACTC) (69).

### Statistics

Statistical analyses were performed using one-way ANOVA with Dunnett’s multiple correction or uncorrected Fishers LSD *t* test, Welch’s unpaired *t* test were performed with Graphpad Prism 10.6 (Graphpad software).

## Declarations

### Ethics approval and consent to participate

Peripheral Blood Mononuclear Cells were isolated from buffy coats of healthy donors (Sanquin, Amsterdam, The Netherlands). Donors gave informed consent, and the study was conducted in accordance with the principles of the Declaration of Helsinki.

## Data availability

All study data are included in the article and supporting information. The manuscript deposited as preprint to BioRxiv: bioRxiv 2025.12.19.695420; doi: https://doi.org/10.64898/2025.12.19.695420.

## Supporting information

This article contains supporting information.

## Patent

WO2024096735.

## Conflict of interest

The authors declare that they have no conflicts of interest with the contents of this article.
