# Supplementary material for: Siglec-1-targeting single domain antibodies block or enhance pathogen binding to dendritic cells
Source: J Biol Chem. 2026 Sep 9;302(10):113542. doi: 10.1016/j.jbc.2026.113542 (PMC13676974; doi:10.1016/j.jbc.2026.113542)
Supplement: Supplementary file 1 [file mmc1.pdf]

Supporting Information

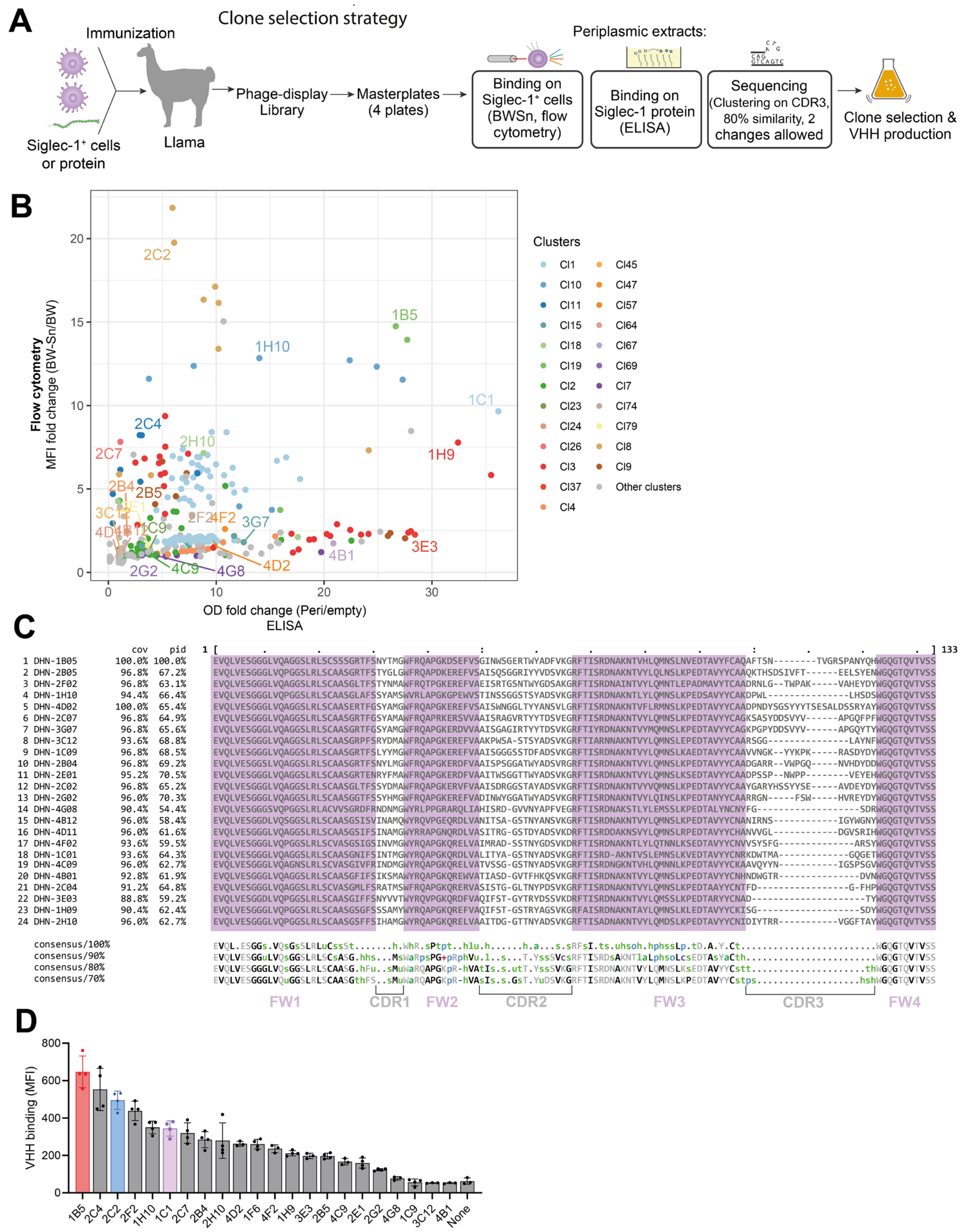

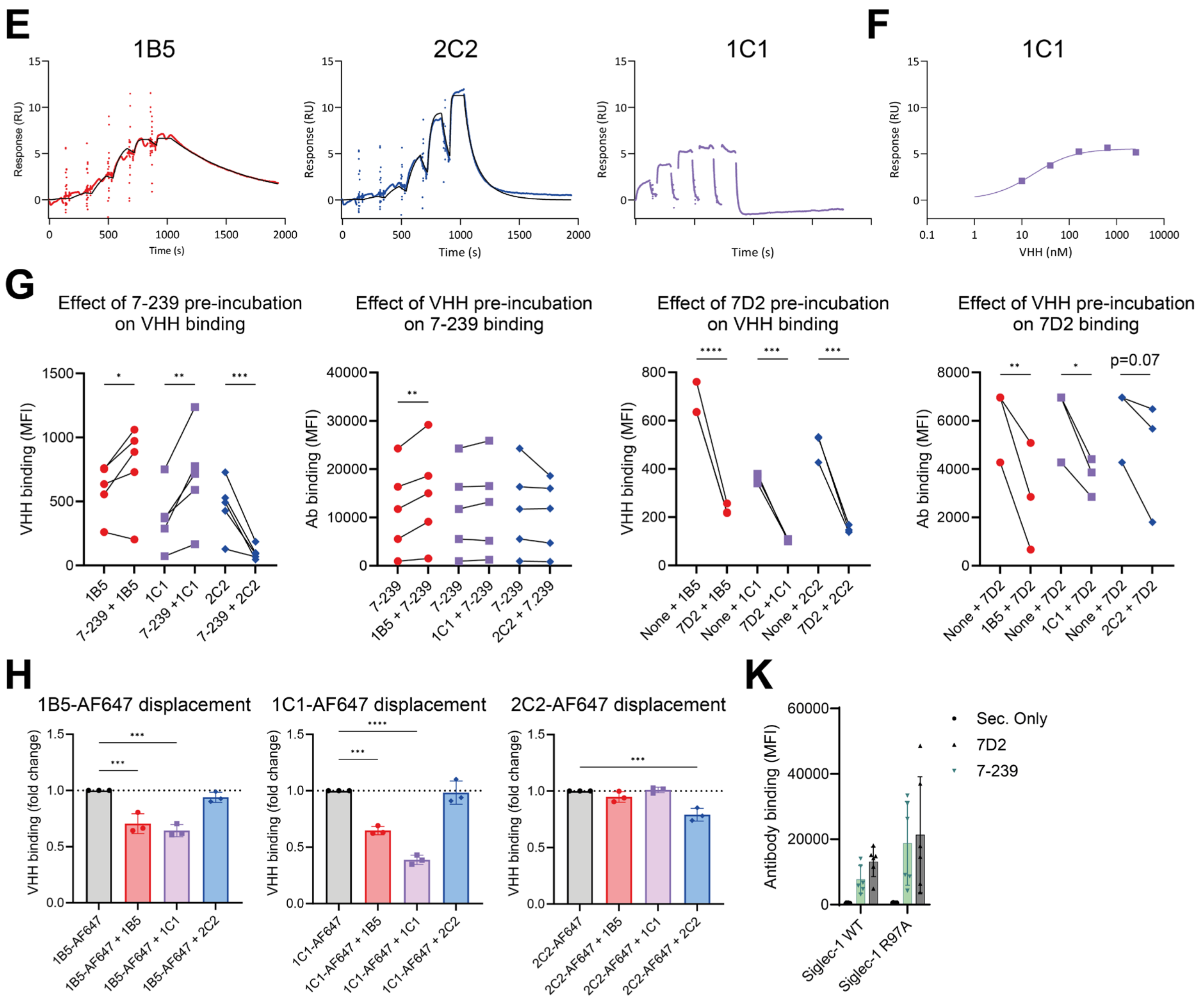

I

VHH/Siglec-1:	2C2/Siglec-1 D1	1B5/Siglec-1 D1-2	1C1/Siglec-1 D1-2
pTM	0.80	0.86	0.80
iPTM	0.86	0.88	0.86

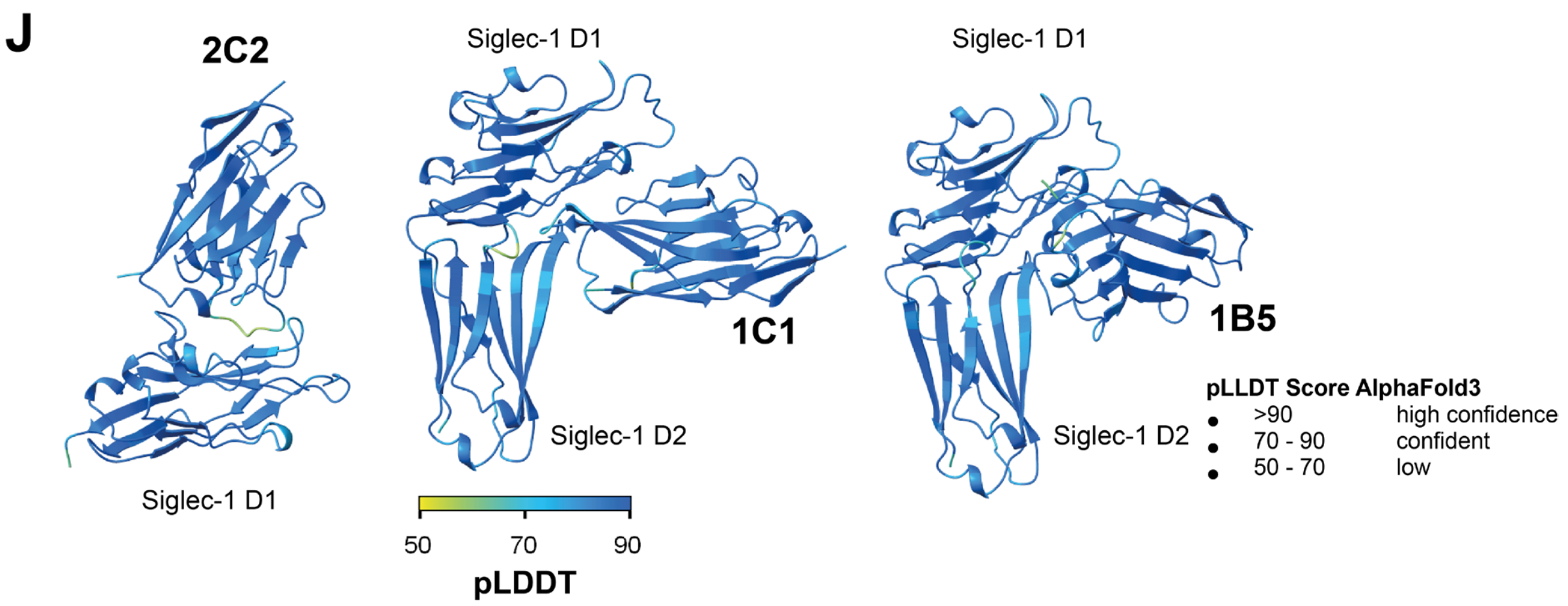

Figure S1 Selection and epitope mapping of Siglec-1-binding VHHs. A) Schematic overview of VHH immunization and clone selection strategy. **B)** VHH periplasmic binding data to (y-axis) BWSn cells, as determined by flow cytometry, or (x-axis) binding to recombinant protein, as determined by ELISA. Selected clones are annotated. **C)** Sequence alignment of selected VHH clones. Framework (FW) regions are highlighted purple flanking complementarity determining regions (CDRs). Consensus sequences are shown below. **D)** VHH binding (500 nM) to Siglec-1 expressing BW5147 cells (BWSn) detected by flow cytometry. Data is shown as geometric mean fluorescent intensity (MFI). Data is shown as mean ± *SD* of n=3 experiments. **E)** Binding of VHH 1B5, 2C2 and 1C1 to immobilized recombinant Siglec-1 (Ser20-Gln-1641) as measured by SPR. Data was fitted using a 1:1 kinetic binding model fit for 2C2 or for 1C1 **F)** show in an R-max plot and fitted for equilibrium binding. **G)** Effect of pre-incubation of BWSn cells with conventional antibody (Ab) 7-239 or 7D2 on VHH binding or the reverse by flow cytometry. Data

is shown as mean fluorescent intensity of at least n=3 experiments (one way ANOVA mixed effect analysis: *p<0.05*,* **p<0.01, ***p<0.001*, ****p*<0.0001). **H)** Binding data of AF647 labelled VHH after competition binding with non-labelled VHH to BWSn cells, data is shown as mean ± *SD* (one way ANOVA: ***p<0.001*, ****p*<0.0001). **I)** Table of predicted template modeling (pTM) and interface predicted modeling (ipTM) scores per fold shown in show in J. **J)** Alphafold3 model of VHH clone 2C2 docked to Siglec-1 domain 1 or VHH clones 1B5 and 1C1 docked to Siglec-1 domain 1-2. Models are colored according to the pLDDT scores with color gradient threshold values set at 50, 70 and 90. **K)** Antibody binding to HEK293T Siglec-1 WT or R97A cells, data is shown as mean ± *SD* of n=3 experiments.

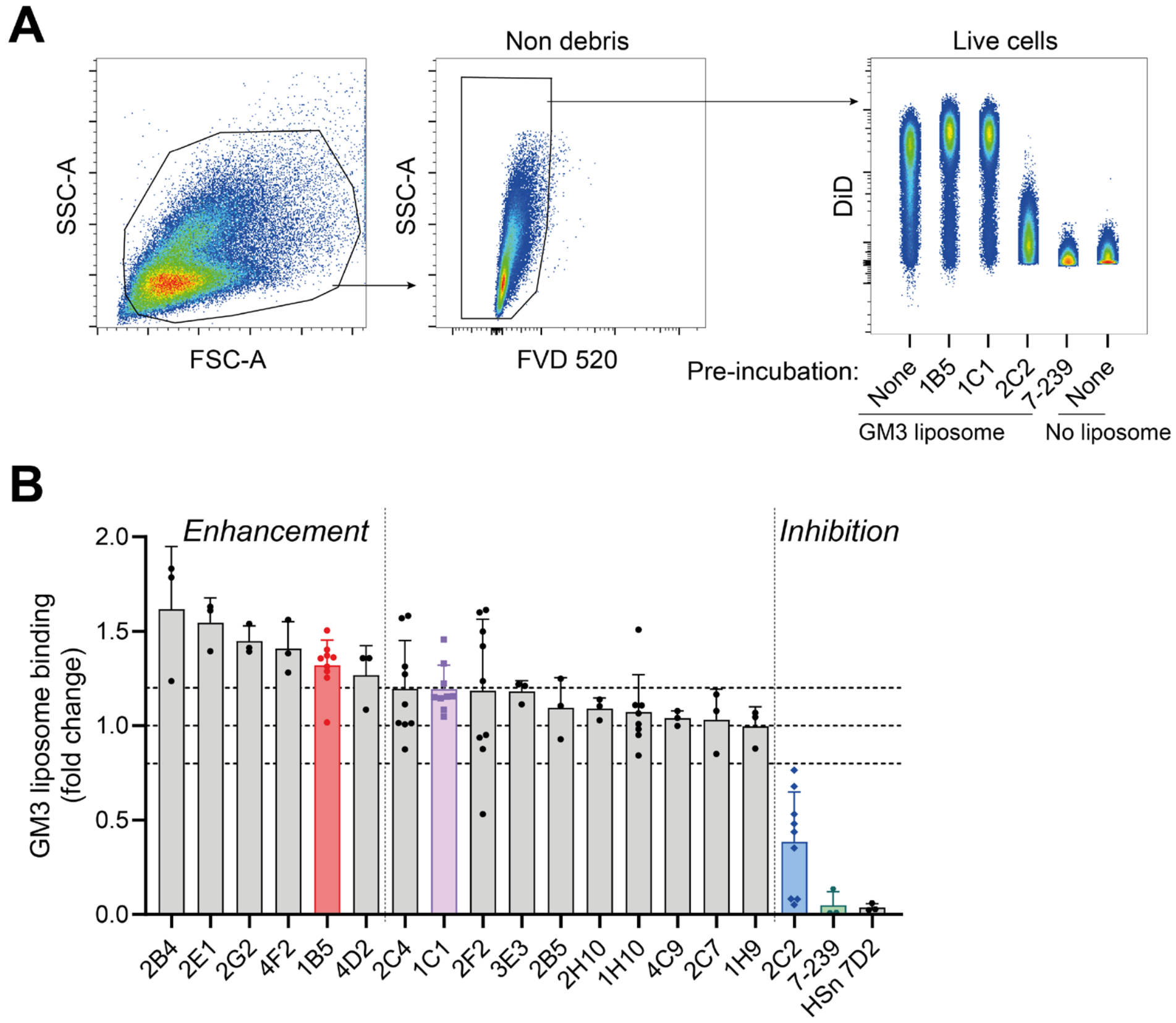

Figure S2 VHH mediated modulation of ganglioside liposome binding to BWSn. A) Gating strategy to determine DiD+ cells after pre-incubation of Siglec-1 expressing BWSn with VHH or conventional antibody followed by incubation with GM3 liposomes. **B)** GM3 liposome binding after VHH or antibody pre-incubation as determined by flow cytometry. Data is normalized to a no VHH control and shown as mean ± *SD* of at least *n*=3 experiments.

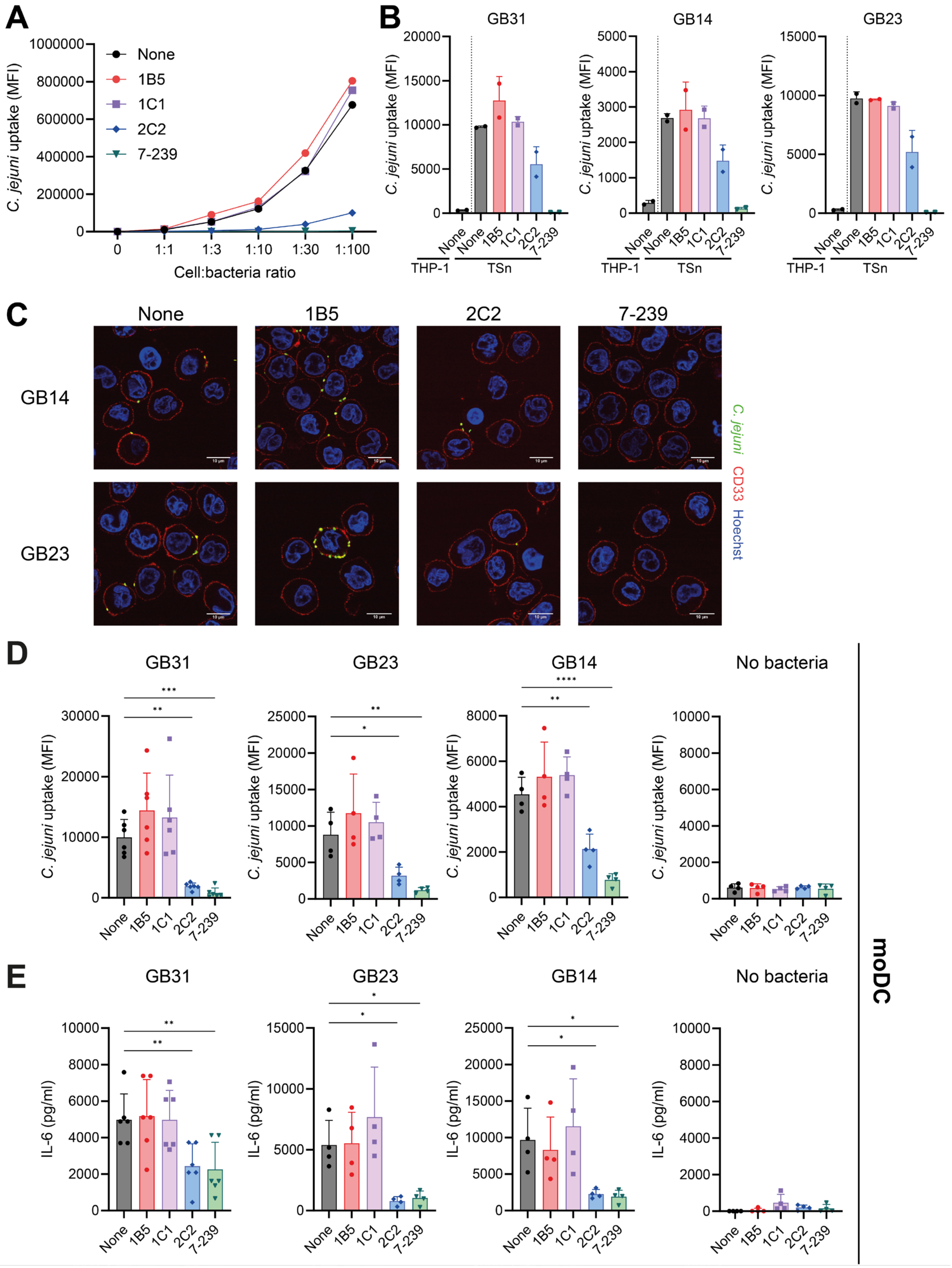

Figure S3 VHH modulate uptake of *C. jejuni*. A) *C. jejuni* strain GB31 uptake by TSn at increasing cell to bacteria ratios after pre-incubation with VHH or antibody. **B)** Uptake of *C. jejuni* strains GB31, GB23, and GB14 by THP-1 and TSn cells pre-incubated with VHH or conventional antibody as determined by flow cytometry. Data is as mean ± *SD* of two experiments. **C)** Fluorescent microscopy images of FITC-labelled GB14 and GB23 *C. jejuni* binding to TSn cells pre-incubated with 7-239 or VHH. TSn membranes detected with anti-CD33-AF647 antibody. Nucleus is stained with Hoechst. **D)** Uptake of *C. jejuni* strains GB31, GB23, and GB14 by moDCs pre-incubated with VHH or conventional antibody as determined by flow cytometry. Data is shown as mean ± *SD* of 4 to 6 donors **E)** IL-6 secretion by moDCs, pre-incubated with VHH or conventional antibody, followed by a 6 hour incubation with *C. jejuni* strains GB31, GB23 and GB14 at 37°C. IL-6 amounts were determined by ELISA. Data is shown as mean ± *SD* of 4 to 6 donors (one way ANOVA: **p*<0.05, ***p*<0.001, *****p*<0.0001).

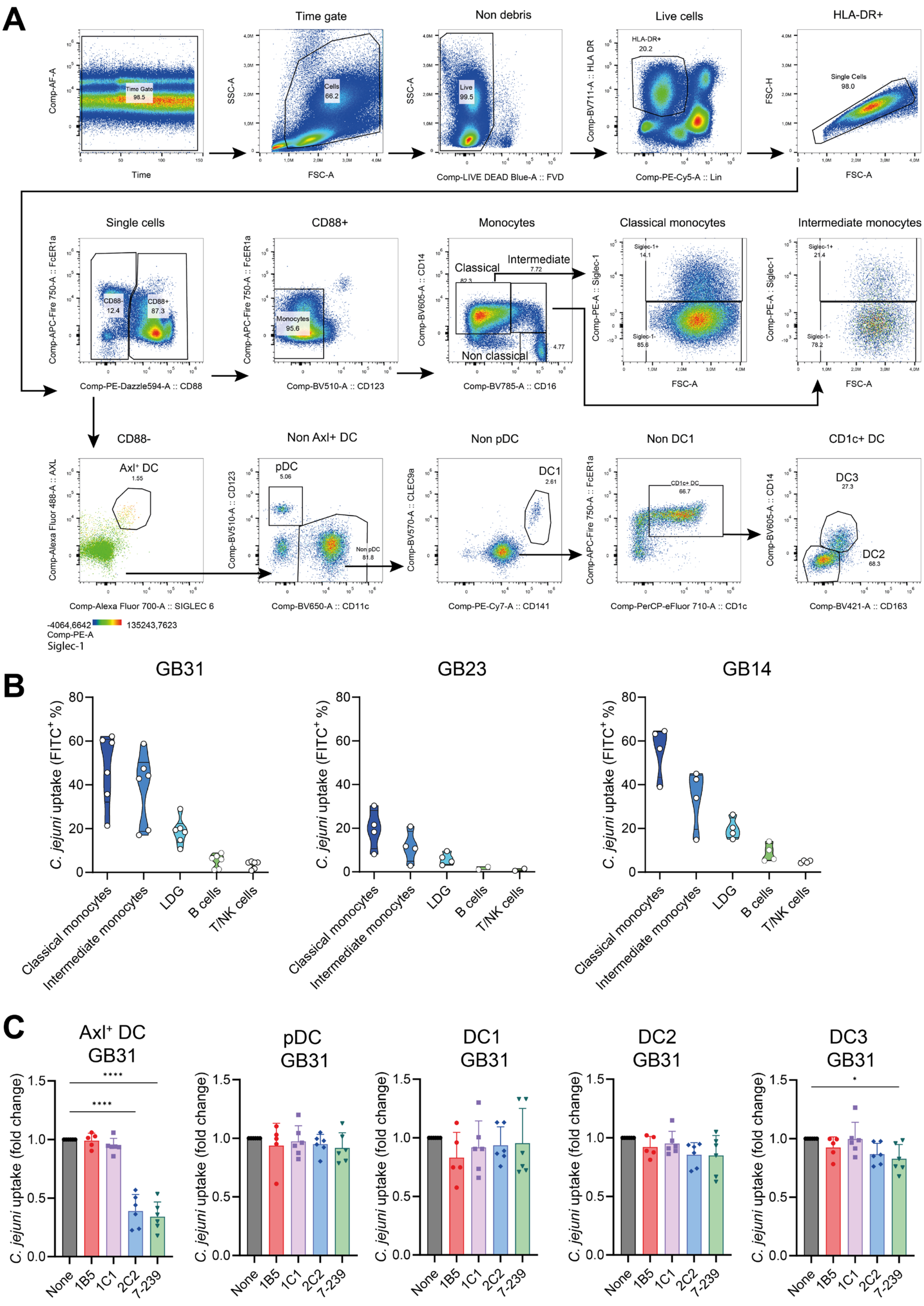
A
Time gate
Non debris
Live cells
HLA-DR+
Comp-AF-A
Time Gate
98.5
Time
SSC-A
Cells
66.2
FSC-A
Live
99.5
Comp-LIVE DEAD Blue-A :: FVD
Comp-BV711-A :: HLA DR
HLA-DR+
20.2
Comp-PE-Cy5-A :: Lin
FSC-H
Single Cells
98.0
Single cells
CD88+
Monocytes
Classical monocytes
Intermediate monocytes
Comp-APC-Fire 750-A :: FcER1a
CD88-
12.4
CD88+
87.3
Comp-PE-Dazzle594-A :: CD88
Monocytes
95.6
Comp-BV510-A :: CD123
Comp-BV605-A :: CD14
Classical
Intermediate
7.72
Non classical
4.77
Comp-BV785-A :: CD16
Comp-PE-A :: Siglec-1
Siglec-1+
14.1
Siglec-1-
85.6
Siglec-1+
21.4
Siglec-1-
78.2
CD88-
Non Axl+ DC
Non pDC
Non DC1
CD1c+ DC
Comp-Alexa Fluor 488-A :: AXL
Axl+ DC
1.55
Comp-Alexa Fluor 700-A :: SIGLEC 6
Comp-BV510-A :: CD123
pDC
5.06
Non pDC
81.8
Comp-BV650-A :: CD11c
Comp-BV570-A :: CLEC9a
DC1
2.61
Comp-PE-Cy7-A :: CD141
Comp-APC-Fire 750-A :: FcER1a
CD1c+ DC
66.7
Comp-PerCP-eFluor 710-A :: CD1c
Comp-BV605-A :: CD14
DC3
27.3
DC2
68.3
Comp-BV421-A :: CD163
-4064,6642
135243,7623
Comp-PE-A
Siglec-1
B
GB31
GB23
GB14
C. jejuni uptake (FITC+ %)
Classical monocytes
Intermediate monocytes
LDG
B cells
T/NK cells
C
Axl+ DC GB31
pDC GB31
DC1 GB31
DC2 GB31
DC3 GB31
C. jejuni uptake (fold change)
None
1B5
1C1
2C2
7-239
****
****
*

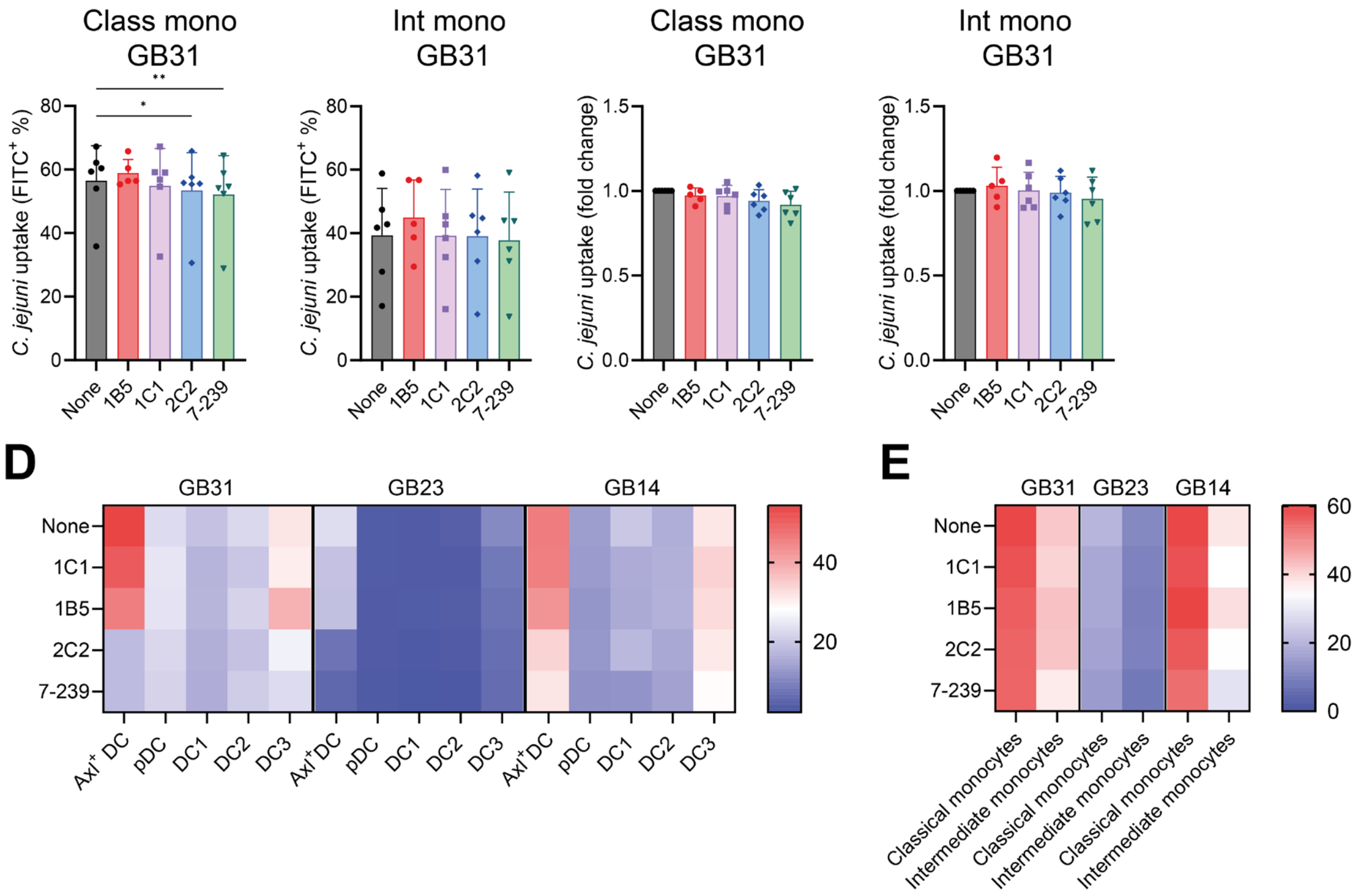

Figure S4 VHH 2C2 blocks uptake of *C. jejuni* by primary cells. A) Conventional gating identifies classical monocytes, intermediate monocytes, plasmacytoid DCs (pDC), conventional DC1 (DC1), conventional DC2 (DC2), DC3, and Axl+ Siglec-6+ DCs (Axl+ DC). **B)** Percentage FITC+ cells after incubation with *C. jejuni* strains GB31, GB23, and 14. Data is shown for 2 to 6 donors. **C)** Uptake of *C. jejuni* strains GB31, GB23, and GB14 by primary cells pre-incubated with VHH or antibody as determined by flow cytometry. Data is normalized to a no VHH control and shown as mean ± *SD* of 4 donors (one way ANOVA: **p*<0.05, ***p*<0.01, *****p*<0.0001). **D)** Heat map of *C. jejuni* strains GB31, GB23, and GB14 uptake by Axl+ DC, pDC, DC1, DC2, and DC3, or **E)** classical (class) or intermediate (int) monocytes after pre-incubation with VHH or conventional antibody shown as % of population positive for indicated *C. jejuni* strain.

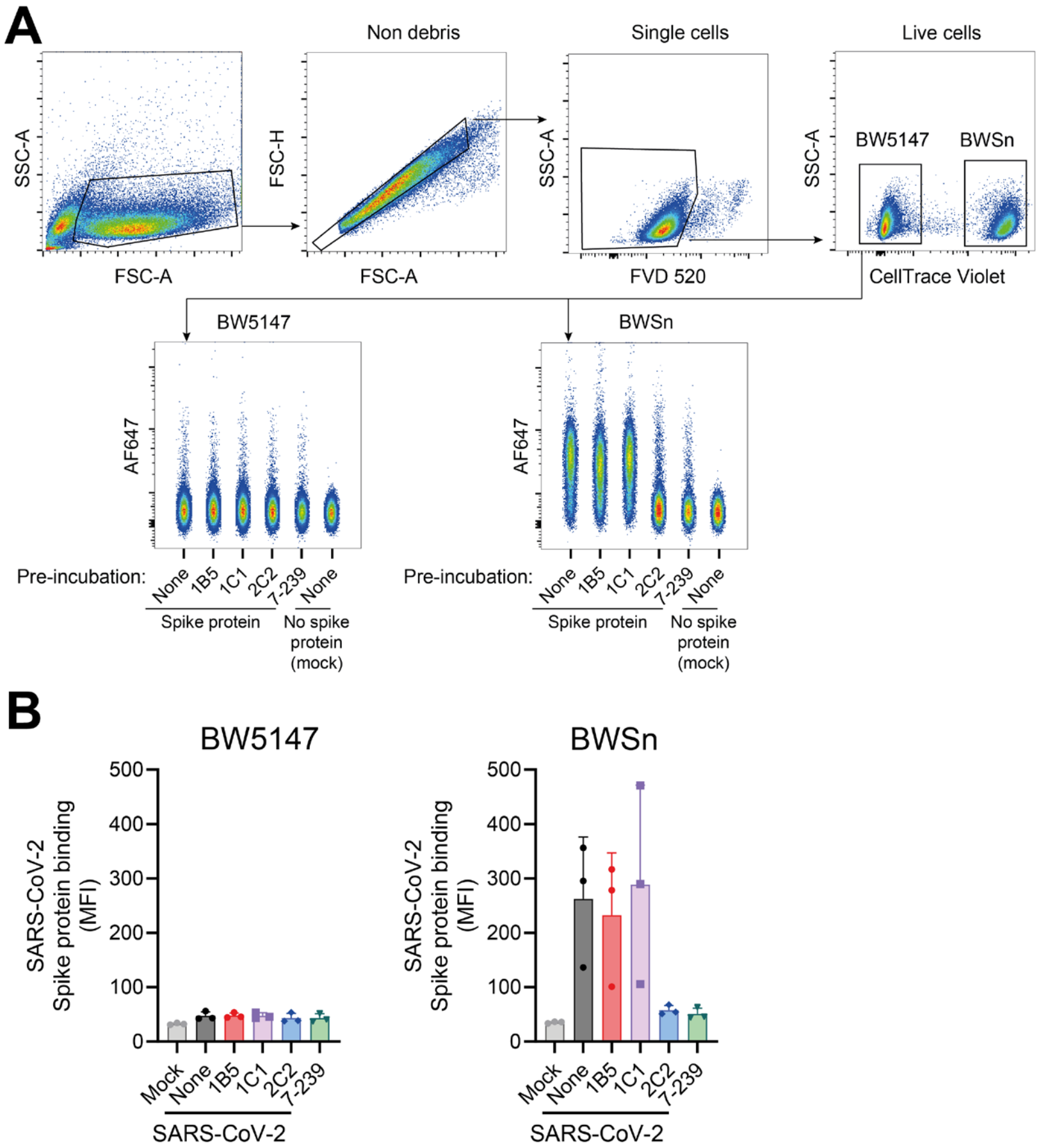

Figure S5 VHH 2C2 blocks SARS-CoV-2 spike protein binding to Siglec-1 A) Gating strategy to identify AF647-labelled SARS-CoV-2 spike protein positive cells by flow cytometry. **B)** SARS-CoV-2 spike protein binding to BW5147 and BWSn cells as detected by flow cytometry. Data is shown as mean ± *SD* of *n*=3 experiments.

Cells	Ligand	Condition	IC_{50} 2C2 (nM)	IC_{50} 7-239 (nM)	Fold difference IC_{50} 7-239/2C2
BWSn	GM3 liposome	4°C, 45 min	87.6 ± 4.8	3.0 ± 1.4	29.7
TSn	GM3 liposome	4°C, 2h	87.8 ± 7.1	5.4 ± 1.4	17.0
TSn	GM3 liposome	37°C, 2h	37.7 ± 2.7	0.8 ± 0.2	51.5
TSn	*C. jejuni* GB31	37°C, 2h	402.0 ± 227.1	0.4 ± 0.1	943.3
TSn	*C. jejuni* GB14	37°C, 2h	666.3 ± 254.7	0.6 ± 0.3	1364.3
TSn	*C. jejuni* GB23	37°C, 2h	522.1 ± 108.5	0.6 ± 0.1	889.7

Table S1 Comparison of 2C2 and conventional antibody 7-239 blocking on binding of GM3 liposome and *C. jejuni*. Data is shown as mean ± *SD* of *n*=3-4 experiments.
